# Exploring the potential of XAI methods in generating clinically meaningful explanations for glycemia prediction in diabetes patients

**DOI:** 10.1186/s12911-026-03420-5

**Published:** 2026-03-14

**Authors:** Sayna Rotbei, Pablo Matías Soler, Beatriz Merino-Barbancho, Laura Lopez-Perez, Arturo Corbatón Anchuelo, Luis Picazo García, Ricardo Mesanza Forés, Laura Mariel Matus, Ricardo Muñoz Albert, Aitor Odiaga Andicoechea, Raquel Piñero Panadero, María Ángeles San Martín Díez, Ainhoa Burzaco Sánchez, Rosana Soriano Barrón, Andrea Irimia, Esther Ruescas Esculano, Mireia Cramp Vinceixo, F. Beddar Chaib, Hania Tourab, Giuseppe Fico, Alessio Botta

**Affiliations:** 1 Department of Electrical Engineering and Information Technology, University of Naples Federico II, Naples, Italy; 2 Emergency Department, Hospital Clínico San Carlos, Madrid, Spain; 3 Life Supporting Technologies Research Group, ETSIT, Universidad Politécnica de Madrid, Madrid, Spain; 4 Servicio de Emergencias Sanitarias Comunidad Valenciana, Valenciana, Spain; 5 Hospital Universitario de Getafe, Madrid, Spain; 6 Hospital de Fuenlabrada, Madrid, Spain; 7 Hospital de la Ribera, Alzira, Spain; 8 Hospital de Galdakao y Hospital de Gernika, Bizkaia, Spain; 9 Clínica Universitaria de Navarra, Navarra, Spain; 10 Hospital de Basurto, Bilbao, Spain; 11 Hospital San Pedro de Logroño, Logroño, Spain; 12 Hospital Universitario Central de Asturias, Oviedo, Spain; 13 Hospital Universitario de Vinalopó, Elche, Spain; 14 Hospital Joan XXIII, Tarragona, Spain; 15 Complejo Asistencial Universitario de Soria, Soria, Spain; 16 Universidad de Valladolid (ES), Valladolid, Spain; 17 Research Institute University Hospital Clínico San Carlos (IdISSC), Madrid, Spain; 18 Spanish Biomedical Research Centre in Diabetes and Associated Metabolic Disorders (CIBERDEM), Madrid, Spain

**Keywords:** Diabetes, Artificial Intelligence, Machine Learning, Hyperglycemia, Hypoglycemia, eXplainable Artificial Intelligence

## Abstract

**Purpose:**

Glycemic emergencies are a frequent cause of hospital admissions and can lead to severe complications, particularly in older or medically complex patients. Anticipating these events is essential for timely intervention and personalized care. This study aimed to identify patients at risk of hypoglycemia or hyperglycemia using routinely collected data from emergency department of 11 hospitals in Spain.

**Methods:**

A comprehensive modeling framework was designed to identify glycemic events from clinical data. Multiple supervised learning algorithms were trained and validated using routinely collected patient variables. Model explainability was ensured through the integration of XAI methods, which quantified the contribution of individual clinical features to prediction outcomes. This approach enabled transparent model behavior, supporting clinical understanding and facilitating patient risk stratification.

**Results:**

The developed models achieved predictive accuracies between 70% and 74%. Explainability analyses revealed distinct glycemic risk patterns: patients aged 87 years and above were predominantly hypoglycemic, while among younger individuals, those with a body temperature exceeding 36 $^{\circ}$C, Chronic Kidney Disease (CKD) (creatinine $> 1.5 \textrm{mg/dL}$), and platelet counts below $200,000 /\mu \textrm{L}$ were more likely to be hyperglycemic, whereas others tended toward hypoglycemia.

**Conclusions:**

These results highlight the predictive value of age, thermoregulation, renal function, and hematologic parameters in assessing glycemic risk. The combination of machine learning and explainability provides interpretable, actionable insights to support early risk stratification and improve outcomes in diabetes care.

**Trial registration:**

This retrospective study was approved by the Comité de Ética de la Investigación con medicamentos (CEIm) of Hospital Clínico San Carlos (Madrid, Spain) (Approval code: 19/332-E). The requirement for informed consent was waived. All procedures followed institutional ethics standards, the Declaration of Helsinki, and applicable national regulations. **Retrospectively registered**.

**Supplementary Information:**

The online version contains supplementary material available at 10.1186/s12911-026-03420-5.

## Introduction

Chronic diseases are persistent health conditions requiring ongoing medical care and can limit daily activities, as noted by the CDC [1] . They contribute to disability, poor health, and increased premature mortality in the EU, with around 550,000 working-age individuals succumbing to these diseases each year, according to the OECD [2]. As the leading cause of mortality, chronic diseases account for the majority of healthcare costs, totaling 115 billion annually, or 0.8% of Gross Domestic Product Commission and Eurostat [3]. Diabetes is a prevalent chronic disease, highlighting the need for awareness and effective management in healthcare initiatives.

Type 1 Diabetes Mellitus is an autoimmune disease characterized by the gradual destruction of pancreatic $\beta$-cells, leading to insulin deficiency and chronic hyperglycemia Annuzzi et al. [4]. Type 2 Diabetes Mellitus is characterized by insulin resistance and impaired glucose metabolism, which can often lead to damage in vital organs, including the heart, kidneys, and eyes Sapra and Bhandari [5]. Effective Diabetes Mellitus management includes insulin therapy, glucose monitoring, diet, and exercise to maintain glycemic control and prevent complications. Postprandial glucose regulation remains particularly challenging in Type 1 Diabetes Mellitus Hoyos et al. [6]. In emergency medical settings, timely intervention is critical for managing diabetic crises. A study involving 237 patients revealed that hyperglycemia was the primary cause of emergency visits. It was observed that individuals with Type 2 Diabetes Mellitus experienced higher rates of admission and longer hospitalization durations, often linked to infections. Conversely, inadequate control of blood glucose levels was identified as the main factor contributing to decompensation in Type 1 Diabetes Mellitus cases Almazán et al. [7].

A groundbreaking advancement in glucose regulation is the artificial pancreas, a sophisticated system designed to replicate the functions of the pancreas. This system integrates a continuous glucose monitor (CGM), which measures glucose levels every five minutes, with an insulin pump that delivers precise doses of insulin through a sophisticated control algorithm Khodaei et al. [8], Annuzzi et al. [9]. While current commercial systems show great potential, they still necessitate manual input for meal data, which limits the extent of automation that can be achieved Annuzzi et al. [10].

Machine Learning has emerged as a powerful tool for managing complex, multidimensional data, enabling the identification of patterns and predictors across various fields—including cybersecurity Botta et al. [11], health diagnostics Peral et al. [12], Rotbei et al. [13], cancer prognosis Rotbei et al. [14], Rotbei et al. [15], and diabetes management Rotbei et al. [16]. In diabetes care, Machine Learning supports personalized glucose regulation by extracting meaningful patterns from CGM data to improve control algorithms and predict blood glucose levels Zhu et al. [17], Jacobs et al. [18]. Techniques like artificial neural networks are especially effective in forecasting glucose trends and preventing hypo/hyperglycemic events Annuzzi et al. [4].

Research on predicting basal insulin requirements is currently limited and frequently concentrates on specific, non-generalizable scenarios Nguyen et al. [19]. Predicting blood glucose levels continues to pose significant challenges due to a variety of influencing factors, such as dietary intake, physical activity, sleep patterns, and emotional states De Bois et al. [20]. Certain machine learning models integrate demographic information and insulin data; however, it is observed that prediction errors significantly increase during mealtime periods Guzman Gómez et al. [21].

A key limitation of existing models is their lack of explainability Annuzzi et al. [4]. This “black-box” nature hinders trust and adoption among clinicians and patients. To address this, eXplainable Artificial Intelligence has gained traction, aiming to make Artificial Intelligence models more transparent by providing human-understandable justifications for predictions Antoniadi et al. [22], Markus et al. [23]. eXplainable Artificial Intelligence methods offer global or local explanations and can be either model-specific or agnostic Annuzzi et al. [4].

**Fig. 1:**
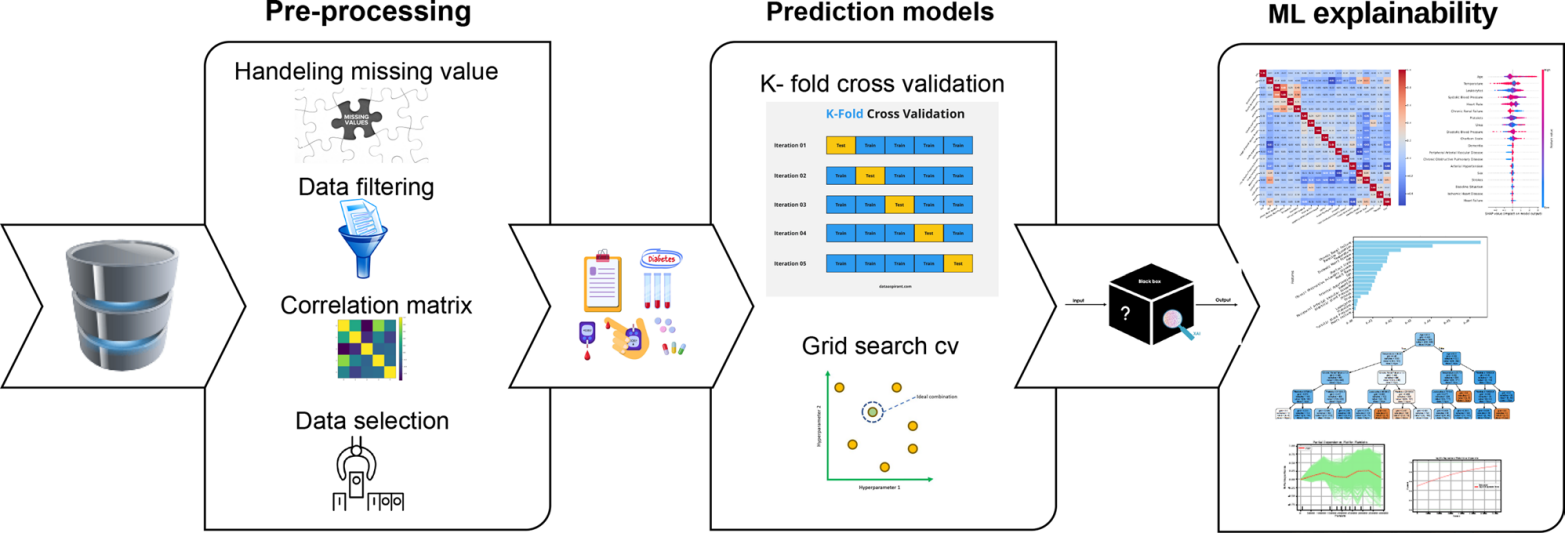
Pipeline of the proposed approach

Accurately predicting glycemic events—both hypoglycemic and hyperglycemic—is a significant challenge in diabetes management. While patient-generated data from continuous glucose monitoring, insulin dosing, and lifestyle tracking are increasing, the variability in individual glycemic responses complicates the development of effective predictive models. These variations stem from various physiological, behavioral, and environmental factors that interact in complex ways, making traditional statistical models less effective in personalized healthcare settings. Our study explores advanced Machine Learning methods combined with eXplainable Artificial Intelligence to enhance the prediction and interpretation of glycemic events. Unlike black-box models with high performance but low transparency, eXplainable Artificial Intelligence techniques reveal the internal reasoning of Machine Learning models, helping to identify factors that influence a patient’s risk. This improves clinical explainability and supports the development of personalized and trustworthy decision-support tools. Our study explores advanced Machine Learning methods combined with eXplainable Artificial Intelligence to enhance the prediction and interpretation of glycemic events. Unlike black-box models with high performance but low transparency, eXplainable Artificial Intelligence techniques reveal the internal reasoning of Machine Learning models, helping to identify factors that influence a patient’s risk. This improves clinical explainability and supports the development of personalized and trustworthy decision-support tools. Our analysis begins with a detailed examination of a dataset comprising 55 distinct variables. To enhance the robustness of our model and mitigate the risk of overfitting, we implemented a comprehensive feature selection process, resulting in a refined subset of 20 key variables. This selection served as the foundation for our predictive modeling efforts. We applied a range of Machine Learning algorithms to forecast glycemic events, supported by various eXplainable Artificial Intelligence techniques. These included correlation matrices, surrogate decision trees, permutation importance, and SHapley Additive exPlanations values, all of which facilitated a clear interpretation of model predictions. This approach allowed us to identify and visualize the principal factors contributing to glycemic risk in a transparent and interpretable manner. To validate our findings, we performed a secondary analysis using the 5 most influential predictors to assess if a simpler model could yield comparable predictive performance. This analysis was enhanced with Partial Dependence Plots and logistic regression models to provide additional insights into the relationship between predictors and glycemic outcomes. Preliminary results indicate that Machine Learning models, even with a minimal feature set, achieve predictive accuracies of 70–74%. More importantly, these models highlight distinct patterns linked to hypo- and hyperglycemic risk profiles, providing insights into individualized glycemic regulation. Our findings emphasize both explainability and clinical relevance, which are crucial contributions to the field. This study introduces a data-driven, explainable framework for predicting glycemic events, combining accuracy and explainability. By utilizing eXplainable Artificial Intelligence, it offers insights into glycemic variability mechanisms, promising advancements in personalized diabetes care, and proactive clinical interventions. Figure 1 provides a clear schematic representation of the proposed method, illustrating its key components and workflow effectively.

## Materials and methods

### Data pre-processing

The dataset referenced in the preceding section required an extensive pre-processing procedure prior to the application of Artificial Intelligence techniques to ensure optimal model performance. To establish a consistent and analytically robust dataset, all records were carefully cleaned, pre-processed, and normalized.

The dataset showed a low incidence of missing values in key predictors, likely due to clinicians overlooking data amid heavy workloads in the emergency department. Missing data accounted for less than 5% per variable and stemmed from both random and systematic factors, making it unlikely to significantly skew the analysis. Missing values were systematically imputed with a value of $-1$, a common practice in emergency department informatics and machine learning. This choice has three advantages: (i) $-1$ does not overlap with any valid physiologic measurements, avoiding ambiguity; (ii) it allows models, especially tree-based algorithms, to identify if the absence of information is informative; and (iii) it reduces data loss compared to listwise deletion, which is critical given the dataset’s modest size. This approach preserves the distinction between observed and missing values, allowing machine learning models to use both as valuable features Josse et al. [24]. Additionally, categorical variables were transformed into numerical representations for compatibility with all algorithms.

Following initial cleaning and imputation, predictors were normalized using *Min-Max scaling*:

$$x\prime = \frac{x - x_{\min}}{x_{\max} - x_{\min}},$$

where $x_{\min}$ and $x_{\max}$ represent the minimum and maximum values calculated from the training data. A comprehensive correlation analysis was subsequently conducted to assess relationships among variables. Predictors exhibiting high collinearity were systematically removed to reduce redundancy, mitigate multicollinearity, and improve the generalizability of the resulting models.

This refined dataset forms the basis for evaluating the impact of the selected predictors on patients’ glycemic status, enabling a more accurate and clinically meaningful analysis of hypoglycemia and hyperglycemia risk in emergency department settings.

### Used ML methods

Following the pre-processing stage, we applied eight different Machine Learning methods to the dataset: Decision Tree (DT), Random Forest (RF), Support Vector Machine (SVM), k-Nearest Neighbors (k-NN), Gradient Boosting (GB), Multi-Layer Perceptron (MLP), Stochastic Gradient Descent (SGD), and AdaBoost. These methods were selected to represent a diverse range of algorithmic families—linear models, tree-based models, instance-based learning, probabilistic methods, ensemble learning, and neural networks—allowing for a comprehensive comparison of their performance on our classification task.

To evaluate the generalizability of each model, we used five-fold cross-validation. This method helps prevent overfitting by splitting the dataset into five equal parts. In each iteration, four folds trained the model while the remaining fold served as the test set. This process was repeated five times, with each fold tested once. The final performance metric was the average across all folds, ensuring a fair comparison of the different algorithms.

### Performance metrics

To evaluate the effectiveness of the Machine Learning algorithms in glycemia level of the patients, four key metrics are considered: precision, recall, F1-score, and accuracy. These metrics are derived from the counts of false positives (*FP*), false negatives (*FN*), true positives (*TP*), and true negatives (*TN*), providing a comprehensive assessment of the model’s performance.

1 $$Accuracy = \frac{TP+TN}{TP+TN+FP+FN}$$

The number of *FP*, *FN*, and *TP* are used for calculating the precision and recall which are defined as follows:

2 $$Precision = \frac{TP}{TP+FP}$$

3 $$Recall = \frac{TP}{TP+FN} $$

As a result of the F1-score, datasets with an imbalanced distribution are more likely to be analyzed better Urtnasan et al. [25]. It can be calculated by using the ratio of recall and precision or *TP*, *FP*, and *FN* as follows:

4 $$F1 = \frac{2*Precision*Recall}{Precision+Recall} = \frac{2*TP}{2*TP+FP+FN}$$

### Explainable Artificial Intelligence

Understanding the role of predictors is essential for accuracy in predictive analytics. To ensure effective model training, the collected data must be relevant and well-structured. Feature selection is crucial in this process, as it enhances model performance and explainability by retaining only the most informative predictors. This may also lead to a reduction in dataset size without compromising prediction quality Chen et al. [26].

We adopted explainable machine learning techniques, or explainable artificial intelligence (XAI), to enhance our understanding of model behavior. These methods clarify how models make predictions, reveal input-output relationships, and highlight key influencing features. By utilizing XAI, we aim for more transparent, accurate, and credible results.

Analyzing the relationship between the target variables and features provides valuable insights, and the importance of each feature dictates the order in which they are considered by the prediction algorithm Rotbei et al. [14]. This paper examines various methodologies used to assess the significance of predictors and their respective functions within the decision-making process. To facilitate a comparative analysis of the influence of different variables, we utilized the permutation method. This technique normalizes the biased measure using a permutation test and produces significance *p*-values for each evaluated feature Altmann et al. [27].

A correlation matrix is used to visualize relationships among variables and the target feature. It helps identify which features are closely associated with the target variable by using colors to represent correlation levels.

A heatmap correlation matrix is a visual representation of the correlation coefficients between multiple variables in a dataset. Correlation measures the strength and direction of a linear relationship between two variables, commonly quantified using Pearson’s correlation coefficient ($\rho$ or $r$), defined as Kijsipongse et al. [28]:

5 $$r_{X,Y}=\frac{\sum_{i=1}^{m}(X_{i}-\bar{X})({\rm Y}_{i}-\bar{{\rm Y}})}{\sqrt{\sum_{i=1}^{m}(X_{i}-\bar{X})^{2}}\sqrt{\sum_{i=1}^{m}({\rm Y}_{i}-\bar{{\rm Y}})^{2}}}$$

where:

6 $$\bar{X} = \frac{1}{m} \sum_{i=1}^{m} X_{i}$$

7 $$\bar{Y} = \frac{1}{m} \sum_{i=1}^{m} Y_{i}$$

The Pearson correlation coefficient is a measure of how two variables are linearly related. The value of $r_{X,Y}$ ranges from $-1$ to $1$. It is close to zero if two instances are uncorrelated. When it is positive, $X$ and $Y$ are correlated. The higher the value, the stronger the correlation. If the value of $r_{X,Y}$ is negative, then $X$ and $Y$ are negatively correlated Kijsipongse et al. [28].

The SHapley Additive exPlanations method is one of the most famous eXplainable Artificial Intelligence techniques used in different areas of research Salih et al. [29]. It has been recognized as an effective tool that can be integrated into Machine Learning workflows to improve the explainability of decisions made by the model. The local interpretation methodology of SHapley Additive exPlanations represents an advancement of the Shapley value concept derived from game theory. This methodology aims to equitably distribute the contributions of participants who collectively realize a specific outcome Li [30]. In the context of machine learning, Shapley values serve to quantify the contribution of each feature within the model that collectively produces a given prediction. The Shapley value for a feature $X_j$ in a model is expressed as follows:

8 $$\textit{Shapley}(X_j) = \sum_{S \subseteq N \setminus \{j\}} \frac{k!(p-k-1)!}{p!} \left[ f(S \cup \{j\}) - f(S) \right]$$

In this context, $p$ represents the total number of features, while $N \setminus \{j\}$ denotes the set of all potential combinations of features excluding $X_j$. The symbol $S$ signifies a subset of features drawn from $N \setminus \{j\}$. The function $f(S)$ indicates the model’s prediction based solely on the features included in $S$, whereas $f(S \cup \{j\})$ reflects the model’s prediction when the feature $X_j$ is incorporated into $S$. The implication of this equation is that the Shapley value of a feature quantifies its marginal contribution to the model’s prediction, averaged across all conceivable subsets of features Li [30].

An additional effective approach to interpreting these models involves transforming Machine Learning methods into interpretable surrogate models, such as decision trees. A decision tree represents a straightforward recursive structure that delineates a sequential classification process. Each tree-based model partitions the dataset multiple times based on various threshold values of the features. At each node, a division of the dataset occurs, resulting in the continuous segmentation into multiple subsets. This process continues until each subset—provided that each leaf within the tree is pure—contains instances originating from a single class exclusively Di Castro and Bertini [31].

The Partial Dependence Plots and logistic regression methods were used to understand the role of every single predictor on the result. Partial Dependence Plots serves as a well-known method for examining the correlation between a specific target variable and a designated set of input features, effectively mitigating the impact of other features Pettorruso et al. [32]. In this method, to gain insights into the influence of individual features on the target variable, it is essential to isolate and visually represent their impact Franklin [33].

The x-axis represents feature values, while the vertical axis shows the partial dependence values, indicating how the predicted target variable changes as the feature on the horizontal axis varies, with other features held constant. The vertical axis scale depends on the target variable’s nature; in classification, it reflects either the predicted probability of the positive class or the actual class label. This method helps identify if a variable has a negative, positive, nonlinear, or no relationship with the target variable.

It is important to emphasize that, due to the models’ compatibility with various explainable artificial intelligence (XAI) methodologies and their effectiveness in handling small datasets, the Gradient Boosting model was selected for the SHAP analysis in this study. The XGBoost algorithm was utilized for the Partial Dependence Plot (PDP) analysis, while the Extra Decision Tree was employed to evaluate feature importance, as depicted in the accompanying bar chart. This combination of models effectively illustrates the contributions of predictors across different methods, particularly when these methodologies demonstrate alignment with one another.

## Experimental results

### Participants

This retrospective multicenter study encompassed subjects admitted to the Emergency Departments of eleven hospitals situated in various geographical regions of northern and central Spain. The cohort comprised patients aged over 18 years who were diagnosed with type I or II diabetes and presented with hyperglycemic or hypoglycemic conditions. These individuals visited the Emergency Departments between July 1, 2018, and July 1, 2019. The research was conducted in compliance with the Declaration of Helsinki, and the study protocol received approval from the Ethics Committee of Hospital Clinico San Carlos on May 6, 2022 (ethical committee code: 19/332-E).

Table 1 shows the main characteristics of the participants in terms of age and gender. According to the table, most of the database information belonged to the older generation. However, the gender distribution was balanced between males and females.

**Table 1:** Participants’ demographic characteristics

Category	Statistic	Value
Age	Mean	71.24
	Median	77.00
	Variance	407.19
	SD	20.18
	Q1	59.50
	Q3	87.00
Sex	Female	697.00
	Male	718.00

The analyzed dataset contains 55 variables that provide extensive information about the patient. This retrospective multicenter analytical and observational study includes demographic, clinical, and blood analysis variables. In total, we had information related to 1415 case studies.

### Experiments pipeline

**Fig. 2:**
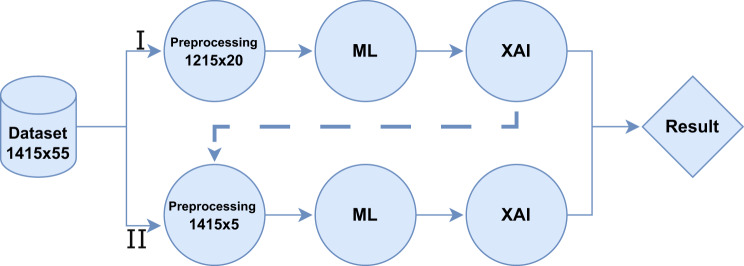
View of the experimental workflow for glycemia prediction in emergency department patients. The process starts with the raw dataset containing 1415 patient encounters and 55 predictors. Two preprocessing pathways are applied: (I) a dimensionality reduction to 20 predictors and (II) a further selection of 5 key predictors. Both preprocessed datasets are subsequently used to train machine learning (ML) models. The outputs of the ML models are then analyzed using explainable AI (XAI) techniques to provide explainability of predictions. Finally, the results from both pipelines were compared to evaluate predictive performance and clinical explainability. Circles represent processing stages (preprocessing, ML, XAI), diamonds represent the final result, and arrows indicate the workflow sequence

For transparency and methodological rigor, we followed the TRIPOD guidelines to ensure comprehensive reporting and validation of our predictive models. To clarify the mechanisms contributing to the opaque nature of Artificial Intelligence and to better understand the impact of various predictors on the glucose levels of patients, we undertook two analytical processes, named *I* and *II*, each comprising a systematic series of steps. Fig 2 shows the pipeline followed to reach the result to achieve the mentioned goals.

In the initial stage of the analytical process, following pre-processing, we conducted a thorough review of the correlation between glucose levels and all variables. To address multicollinearity, predictors with a Pearson correlation coefficient of 0.5 or higher were removed, eliminating 35 variables due to their strong correlation with the glycemic event and with each other. This helps reduce potential bias. The cutoff of $|r| \geq 0.5$ is a standard in statistical modeling, indicating a moderate-to-strong linear relationship where variables share at least 25% of their variance. This cutoff balances preserving valuable information and maintaining model stability. A full list of the eliminated variables is available in Table 2. Additionally, 200 cases were excluded from the study due to a substantial percentage of missing values among the 20 variables that remained under consideration. In the dataset, there are a total of 318 cases identified with hyperglycemia and 897 cases identified with hypoglycemia as it showed in the Table 3 .

**Table 2:** Variables excluded from the analysis

Variable	Variable
Emergency care date	Previous Metformin treatment
Blood glucose level upon arrival at Emergency Department	Previous Sulfonylurea treatment
Dyslipidemia	Previous Meglitinide treatment
Diabetes Mellitus	Previous Thiazolidinedione treatment
Previous GLP-1 analogue treatment	
Previous SGLT2 inhibitor treatment	
Antidiabetic treatment before admission	Previous combination antidiabetic treatment
Previous long-acting basal insulin	Previous ultra-long-acting basal insulin
Previous short-acting insulin	Previous faster-acting insulin
Previous intermediate-acting basal insulin	Previous mixed insulin
Previous insulin therapy	DM treatment upon discharge
Metformin upon discharge	Sulfonylureas upon discharge
Meglitinides upon discharge	Thiazolidinedione upon discharge
DPP-4 inhibitor upon discharge	SGLT2 inhibitors upon discharge
Long-acting basal insulin upon discharge	Ultra-long-acting basal insulin upon discharge
Short-acting insulin upon discharge	Faster-acting insulin upon discharge
Intermediate-acting basal insulin upon discharge	Mixed insulin upon discharge
Insulin therapy upon discharge	Glomerular filtration
Creatinine

**Table 3:** Phase I: summary of glycemic events in the dataset

Condition	Number of Cases
Hyperglycemia	318
Hypoglycemia	897

After studying the dataset, eight different ML algorithms were applied and their performances compared to identify the best predictor for patient glycemia levels. Following this evaluation, the next step was to examine the rationale behind the models’ decisions by implementing the Explainable AI methods discussed previously. Exploring the complexities of machine learning (ML) reveals that achieving high performance often requires creating tailored profiles for different cases. However, this profiling approach has two main drawbacks. First, methods may not generalize well, leading to decreased effectiveness in new situations. Second, the algorithms’ opaque behavior can complicate understanding and interpreting their decision-making processes, undermining trust in ML outcomes, especially in critical applications.

It should be highlight that although baseline classifiers or simple heuristic rules were not evaluated in this study, we ensured that the models leveraged clinically meaningful features through explainability analyses. XAI based methods attribution confirmed that the predictors with the highest influence—such as age, renal function, body temperature, and platelet count—are well aligned with clinical expectations for glycemic risk. This alignment supports the clinical credibility of our models and suggests that their performance derives from capturing complex, multivariable relationships rather than spurious associations or simple single-variable thresholds. Future work may include formal comparisons with naïve or rule-based baselines to further quantify the added value of multivariable modeling.

To address the identified challenges, the dataset underwent a refinement process, focusing on five key predictors for analysis. This strategic choice allowed for the exclusion of other variables, preserving the integrity of all cases, which totaled 1415, including 430 instances of hyperglycemia and 985 of hypoglycemia As it showed in the Table 4.

**Table 4:** Phase II: summary of glycemic events in the dataset

Condition	Number of Cases
Hyperglycemia	430
Hypoglycemia	985

Subsequently, Machine Learning and eXplainable Artificial Intelligence methods were used to predict glucemia events and understand the influence of the five selected predictors. While this narrowed focus reduced the accuracy of the ML methods by about 5% points, it significantly improved generalizability and the clarity of explanations, making the findings more interpretable for users.

### Machine learning I: wide feature set

In the initial phase of the experimental study, we implemented eight distinct Machine Learning algorithms on a dataset comprised of 20 predictors and 1215 cases. The results of our analysis are summarized in Table 5, which illustrates the performance metrics for each Machine Learning method applied. Upon careful examination of the data presented, it is evident that all the employed Machine Learning techniques achieved remarkably similar outcomes, with performance variations typically limited to only a few percentage points, particularly in terms of accuracy and precision.

**Table 5:** Performance comparison of various Machine learning models

Model	Precision	Recall	F1 Score	Accuracy
Random Forest	0.65	0.73	0.64	0.73
SVM	0.55	0.74	0.63	0.74
KNN	0.64	0.71	0.65	0.71
Decision Tree	0.60	0.73	0.63	0.73
MLP	0.67	0.74	0.63	0.74
SGD	0.55	0.74	0.63	0.74
AdaBoost	0.67	0.74	0.63	0.74
Gradient Boosting	0.59	0.74	0.63	0.74

Table 6 presents a summary of the performance metrics for various machine learning models in the identification of hypo- and hyper-condition cases. The Random Forest and Gradient Boosting algorithms exhibit strong performance, demonstrating moderate sensitivity to hyper-conditions (0.385 and 0.437, respectively) and high sensitivity to hypo-conditions (0.92 and 0.896). The Decision Tree method achieves high sensitivity for hypo-conditions (0.959), yet its sensitivity for hyper-conditions is notably low (0.174). Stochastic Gradient Descent (SGD) accurately identifies hypo-conditions (1.0) but is ineffective in detecting hyper-conditions (0.0), resulting in undefined Positive Predictive Value (PPV) and Negative Predictive Value (NPV) metrics. Both the Support Vector Machine (SVM) and K-Nearest Neighbors (KNN) models demonstrate moderate performance; the SVM model exhibits low sensitivity for hyper-conditions (0.207) while maintaining good specificity. In conclusion, Gradient Boosting and Random Forest provide the most effective balance in detection capabilities for both hypo- and hyper-conditions.

**Table 6:** Phase I: performance metrics for different models

Model	**Sens**$-hypo$	**Sens**$-hyper$	**Spec**$-hypo$	**Spec**-$hyper$	**PPV**$-hypo$	**PPV**-$hyper$	**NPV**$-hypo$	**NPV**-$hyper$
RF	0.92	0.385	0.385	0.92	0.775	0.682	0.682	0.775
SVM	0.925	0.207	0.207	0.925	0.728	0.555	0.555	0.728
KNN	0.847	0.337	0.337	0.847	0.745	0.491	0.491	0.745
DT	0.959	0.174	0.174	0.959	0.762	0.704	0.704	0.762
MLP	0.854	0.356	0.356	0.854	0.752	0.520	0.520	0.752
SGD	1.000	0.000	0.000	1.000	0.696	—	—	0.696
AB	0.908	0.330	0.330	0.908	0.756	0.649	0.649	0.756
GB	0.896	0.437	0.437	0.896	0.787	0.660	0.660	0.787

Notably, among the diverse methods tested, both Decision Tree and Multi-Layer Perceptron exhibited superior performance, establishing themselves as the top contenders in this evaluation. It should be mentioned that to achieve this performance, the GridSearchCV technique was used in order to hyper-tune the parameters of every algorithm.

The Multi-Layer Perceptron is a powerful supervised learning algorithm designed to approximate complex functions by leveraging training data. In this context, let $d$ represent the number of input dimensions, while $o$ signifies the number of output dimensions. The model processes a set of features represented as $\mathbf{x} \in \mathbb{R}^d$ and a corresponding target output $\mathbf{y} \in \mathbb{R}^o$. Through the training process, the Multi-Layer Perceptron is capable of learning intricate non-linear relationships, making it suitable for tasks involving either classification—where the goal is to categorize inputs into distinct classes—or regression- where it predicts continuous values. Unlike Logistic Regression, which essentially provides a linear decision boundary, the Multi-Layer Perceptron incorporates one or more non-linear layers interposed between the input and output layers. These intermediary layers, known as hidden layers, enable the model to capture complex patterns within the data, enhancing its overall predictive power and versatility across a variety of applications. MLP effectively captures nonlinear interactions among clinical features.

An Decision Tree classifier is a sophisticated meta-estimator designed to enhance classification performance. It initiates the process by fitting a classifier to the original dataset, establishing a baseline model. Subsequently, it iteratively refines the model by training additional classifiers on the same dataset, with a crucial adjustment: the weights of instances misclassified by previous classifiers are increased. This strategic reweighting ensures that subsequent classifiers focus on the more challenging cases, thereby improving the ensemble’s accuracy and robustness in class discrimination. Through this iterative learning approach, Decision Tree adeptly transforms weak learners into a powerful predictive model. AdaBoost sequentially emphasizes difficult-to-classify instances, improving robustness

Although we did not explore ensembles combining these models, future work could investigate stacking or voting approaches to enhance predictive accuracy, potentially.

### Explainable AI I: wide feature set

**Fig. 3:**
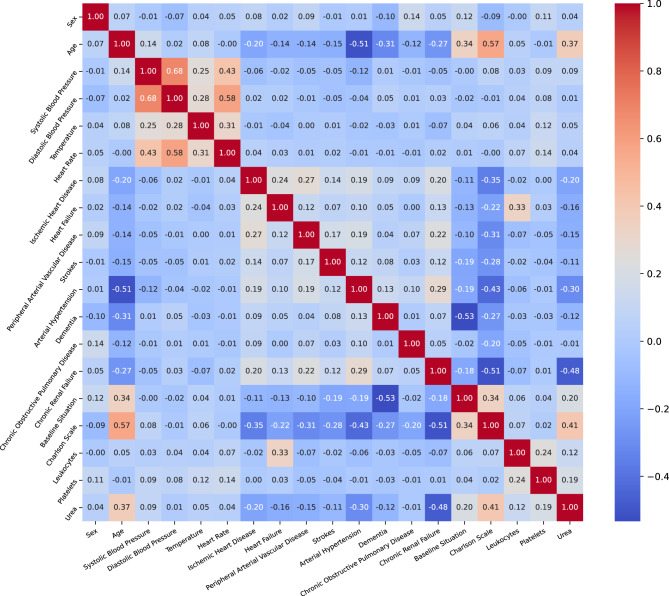
Heatmap showing the correlation coefficients between all variables in the dataset, illustrating the strength and direction of linear relationships

In this phase, we conducted a comprehensive analysis of the dataset to gain deeper insights into the influence of various predictors. To achieve this, we initially employed a correlation matrix, a powerful tool that allowed us to evaluate and identify the relationships between all the predictors systematically. This matrix provided a clear visual representation of how each variable interacts with one another, shedding light on potential patterns and associations that could inform our understanding of the dataset. Fig 3 illustrates the intricate correlations among all variables present in the dataset. Analyzing this figure reveals that certain predictors, such as systolic blood pressure, diastolic blood pressure, and heart rate, exhibit a strong interdependence. This suggests that a variation in the value of one of these predictors is likely to induce corresponding changes in the others, highlighting the complex dynamics at play within the cardiovascular metrics.

**Fig. 4:**
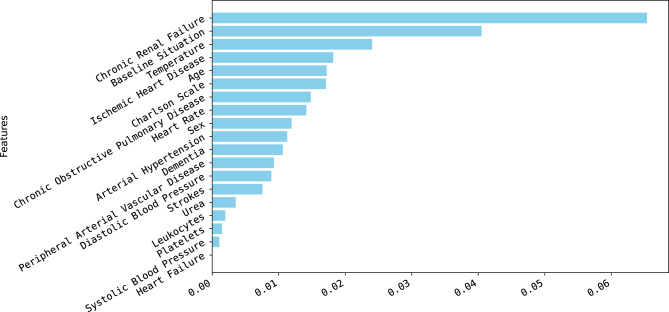
Importance of all variables involved in the analysis

For the next phase of the analysis, we assessed the significance of the predictors in the Machine Learning classification problem using two distinct methodologies. The first approach employed was the permutation method, which effectively illustrates the impact of each variable on the overall outcome. This method allows us to understand how each predictor influences the final results. The second approach utilized was SHapley Additive exPlanations, a powerful technique that provides deeper insights into the contribution of each feature, enabling a more nuanced understanding of their roles in the model’s predictions. Together, these methods offer a comprehensive evaluation of the predictors, highlighting their importance in the classification task. Figure 4 illustrates the significant impact of various predictors on the final decision-making process. The analysis reveals that the top five most influential factors are chronic renal failure, the patient’s baseline health condition, body temperature, ischemic heart disease, and age. This highlights that the Machine Learning algorithms prioritize information derived from these variables over others, such as heart failure, when determining the likelihood of a patient’s glycemic event. These findings underscore the critical role that specific health indicators play in guiding clinical decisions in the context of glycemic management.

**Fig. 5:**
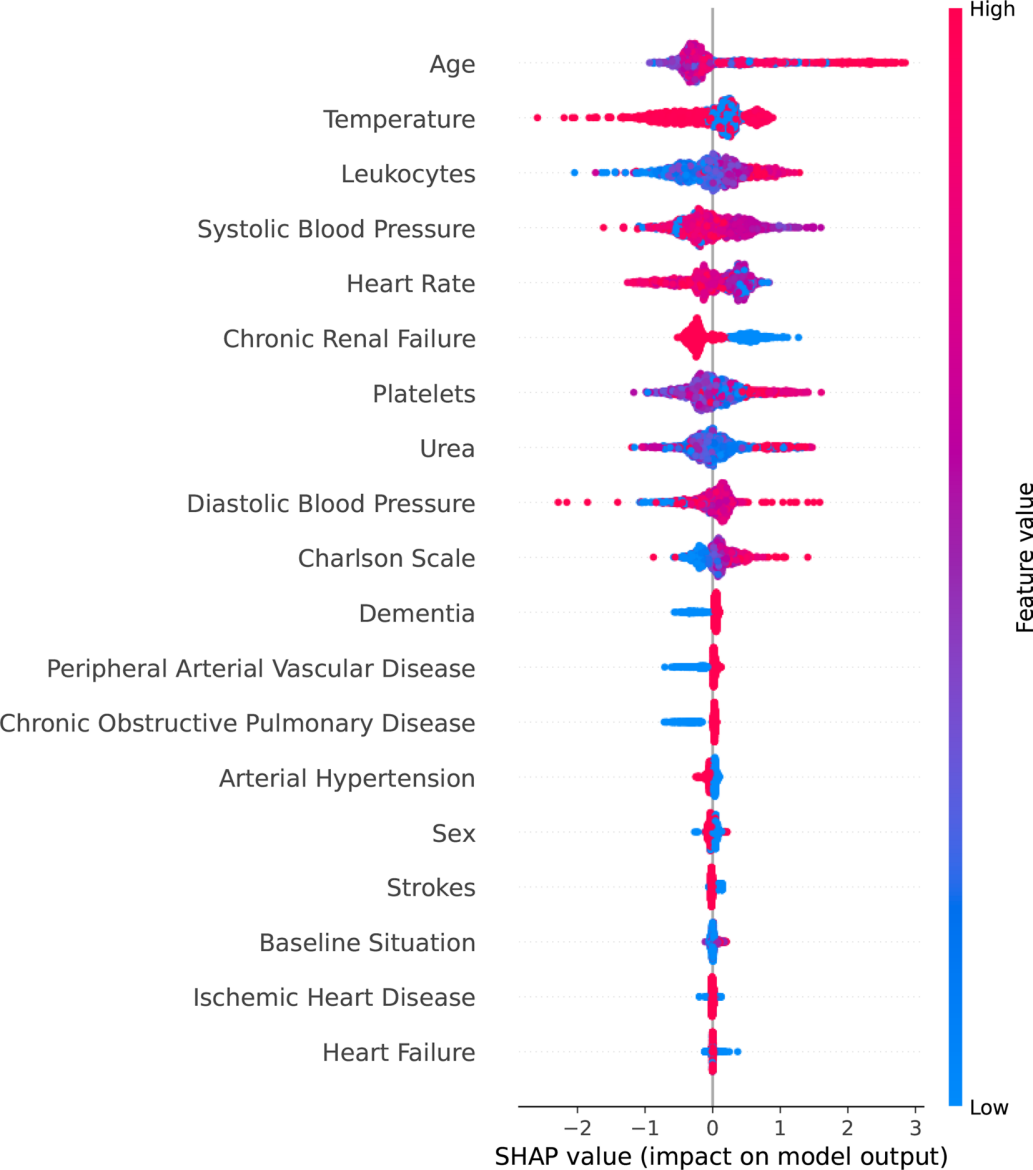
SHAP values for the Gradient Boosting model, showing how each predictor influences the model’s predictions and highlighting their relative importance in the dataset

Figure 5 illustrates the distribution of SHapley Additive exPlanations values, providing valuable insights into the significance of various variables in the predictive model. This plot not only highlights the importance of individual features but also demonstrates their collective impact on the prediction for each case. For instance, it is evident from the plot that an increase in age tends to have a positive influence on the predicted outcome, suggesting that older individuals are associated with higher predictions. In contrast, the data indicates that rising temperatures could adversely affect the final results, emphasizing the nuanced interplay between these variables in the modeling process.

In the subsequent phase, a surrogate decision tree was employed to elucidate the intricacies of the black-box nature of Machine Learning algorithms. Figure 1 of the supplementary material presents the surrogate decision tree derived from the current analysis, illustrating the intricate relationships and decision-making pathways identified in the data. This visual representation highlights key variables and their influence on the overall model, offering a clearer understanding of how different factors contribute to the outcomes observed in the analysis. Utilizing this surrogate model, it was demonstrated that the Machine Learning methods applied to the current dataset are predominantly focused on constructing a generalized profile that captures the majority of cases. The process involves establishing various thresholds to determine whether a patient’s condition qualifies as hyperglycemia or hypoglycemia. This careful delineation helps in accurately categorizing the patients.

### Machine learning II: core feature set

In the next phase of the analysis, the influence of each predictor variable was aimed to be elucidated, along with how their individual values were understood to contribute to the final decision-making process. Building on the insights that had been garnered from the previous section, exclusive focus was placed on the top five most significant features. This strategic choice was made not only to enhance the transparency of the decision-making but also to bolster the explainability of the methodologies. Furthermore, by narrowing the focus, the risk of overfitting was mitigated, thereby enhancing the generalizability of the model. Table 7 illustrates the performance of the various Machine Learning methods employed in our study. This table highlights several noteworthy observations. Notably, despite the limited number of variables utilized, the Multi-Layer Perceptron and Decision Tree algorithms demonstrate superior performance compared to their counterparts. Additionally, it is significant to note that even a modest reduction of 4–5% points in performance indicates that the five most critical predictors provide sufficient information, enabling us to achieve a commendable accuracy rate of up to 70%. This finding underscores the importance of analysing key variables to get better insight about Machine Learning methods.

**Table 7:** Performance of utilized ML methods using 5 key predictors

Model	Precision	Recall	F1 Score	Accuracy
Random Forest	0.52	0.69	0.57	0.69
SVM	0.49	0.70	0.57	0.70
KNN	0.57	0.64	0.58	0.64
Decision Tree	0.49	0.69	0.57	0.69
MLP	0.49	0.70	0.57	0.70
SGD	0.49	0.70	0.57	0.70
AdaBoost	0.49	0.70	0.57	0.70
Gradient Boosting	0.49	0.70	0.57	0.70

Table 8 presents performance metrics for various machine learning models in detecting hypo- and hyper-conditions. Random Forest has high Sens-hypo (0.888) but moderate Sens-hyper (0.356). SVM shows the highest Sens-hypo (0.969) with very low Sens-hyper (0.079), indicating a bias toward hypo-cases. KNN and Decision Tree perform moderately, while MLP has high Sens-hypo (0.94) but low Sens-hyper (0.128). SGD detects all hypo-cases (Sens-hypo 1.0) but fails with hyper-cases. Ensemble methods like AdaBoost and Gradient Boosting achieve balanced results, with Sens-hypo around 0.9 and Sens-hyper 0.3–0.34, illustrating the trade-offs between sensitivity and specificity in predicting hyper-conditions.

**Table 8:** Phase II: performance metrics for different models

Model	Sens-hypo	Sens-hyper	Spec-hypo	Spec-hyper	PPV-hypo	PPV-hyper	NPV-hypo	NPV-hyper
RF	0.888	0.356	0.356	0.888	0.76	0.599	0.599	0.76
SVM	0.969	0.079	0.079	0.969	0.707	0.574	0.574	0.707
KNN	0.811	0.402	0.402	0.811	0.757	0.487	0.487	0.757
DT	0.872	0.272	0.272	0.872	0.735	0.487	0.487	0.735
MLP	0.94	0.128	0.128	0.94	0.712	0.521	0.521	0.712
SGD	1.0	0.0	0.0	1.0	0.696	—	—	0.696
AB	0.902	0.288	0.288	0.902	0.744	0.587	0.587	0.744
GB	0.899	0.342	0.342	0.899	0.758	0.615	0.615	0.758

### Explainable AI II: core feature set

**Fig. 6:**
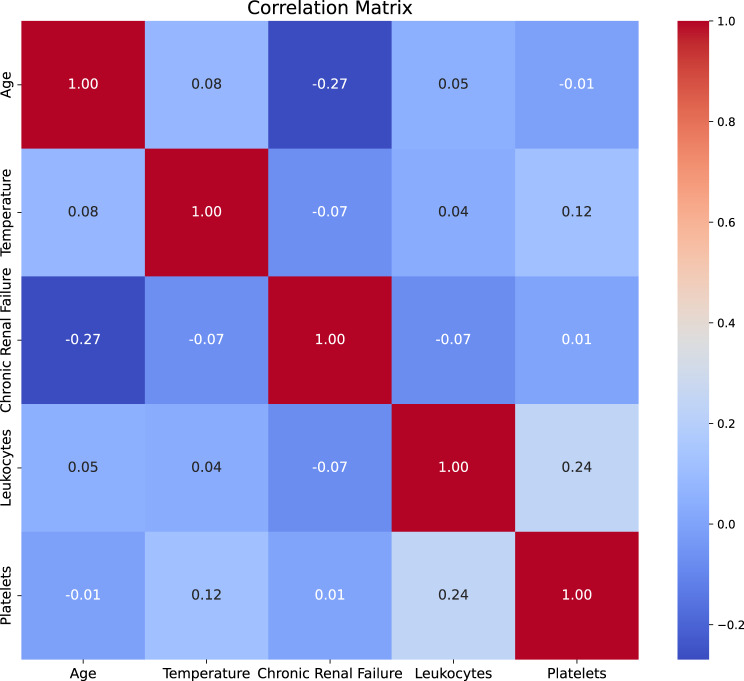
Heatmap showing the correlation coefficients between five key predictors in the dataset, to quantify the linear relationships between variables

Figure 6 illustrates the relationships among five key variables, demonstrating that none exhibit a high degree of correlation with one another. This indicates that each variable contributes unique information to the Machine Learning algorithms employed in our analysis. This unique contribution could explain why, despite removing 15 variables from the dataset, we still achieved an impressive performance level of up to 70%.

**Fig. 7:**
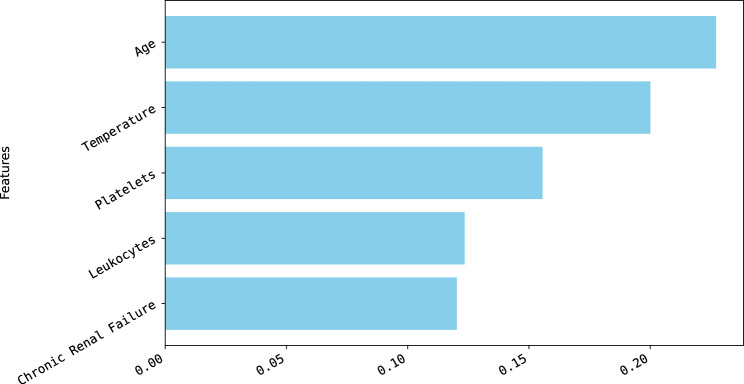
Importance of key predictors

In the subsequent step of the analysis, the permutation method was employed to determine if the ranking of key predictors, regarding their impact on the final decision, experienced any alterations. As shown in Fig 7, despite the dataset being constrained to only 5 predictors, both the significance and the order of these crucial variables remained unchanged throughout the evaluation.

**Fig. 8:**
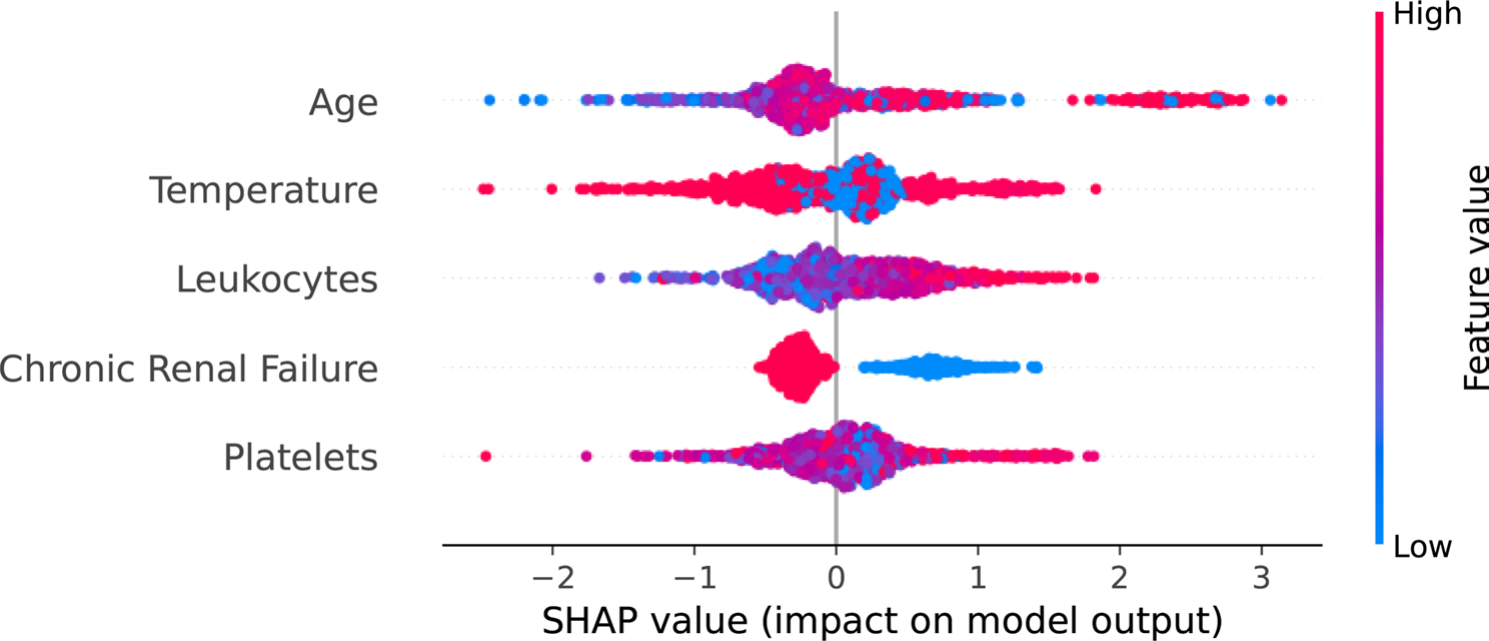
SHAP values for key predictors in the Gradient Boosting model, showing the impact of each feature on the model’s predictions and their relative importance

To conduct a comprehensive exploration and analysis of the behavior of key predictors and evaluate the impact of varying values, we present the summary plot of SHapley Additive exPlanations values, as illustrated in Figure 8. The results depicted in the plot indicate that older individuals (red points) have a positive influence on the final decision, whereas the blue points representing the younger generation exhibit a negative impact on the decision-making process. Furthermore, concerning participants with chronic renal failure, it is observed that high values associated with renal failure contribute negatively, while lower values exhibit a positive contribution.

To enhance understanding and shed light on the opaque nature of Artificial Intelligence, the subsequent steps involved the visualization of surrogate decision trees, Partial Dependence Plots, and logistic regression models. These illustrations serve as valuable tools, enabling us to discern the decision thresholds more clearly. By examining these plots, we can gain deeper insights into the decision-making processes of the Artificial Intelligence system.

**Fig. 9:**
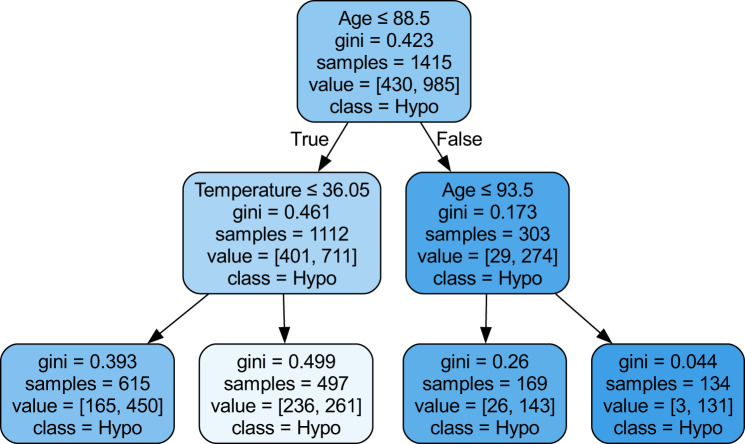
Simplified three-layer surrogate decision tree used to explain black-box model behavior

Based on the information from Fig 9, the darker condition boxes and low Gini coefficient signifies a higher degree of certainty in the classification outcomes. Consequently, the algorithm exhibits enhanced competency in differentiating between hypoglycemia and hyperglycemia, thereby increasing the reliability of its diagnostic capabilities. The first decision point was made based on the age, which is also recognized by other methods as a key predictor. An incorrect assessment indicates that participants are older than 87 years. This threshold is also recognizable from the Partial Dependence Plots and logistic regression plots related to the age, which were shown in Figure 2 of the supplementary material. Figure 9 shows key split thresholds and class distributions used to predict Hypo outcomes; the complete versions of all three decision trees are provided in the supplementary material.

**Fig. 10:**
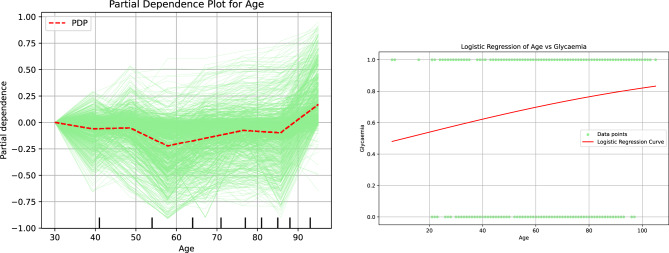
Partial dependence and logistic regression analyses highlighting the role of age in patients’ hypo- or hyperglycemia

The analysis of the Partial Dependence Plots plot in Fig 10, reveals a significant shift in decision-making patterns that occurs after the age of 87. This finding not only aligns with the insights gleaned from surrogate decision trees but also underscores the importance of this age threshold as a critical factor in assessing the potential risk of hypoglycemia. The data compellingly illustrates that individuals beyond this age may require heightened attention and care regarding their blood sugar levels.

The analysis of the logistic regression plot related to age reveals that, although there is no significant shift in the decision-making threshold, there is a definitive correlation indicating that the likelihood of being diagnosed with hypoglycemia increases with age. This finding underscores the importance of considering age as a relevant risk factor in the assessment of hypoglycemia.

The data presented in Fig 9 reveals a crucial decision-making juncture centered on the measurement of body temperature. This figure delineates two distinct thresholds, specifically at 36$^{\circ}$C and 37$^{\circ}$C, and underscores a notable relationship between hyperglycemia and elevated body temperature. This correlation suggests that patients who are experiencing hyperglycemia are significantly more likely to also exhibit a heightened body temperature.

**Fig. 11:**
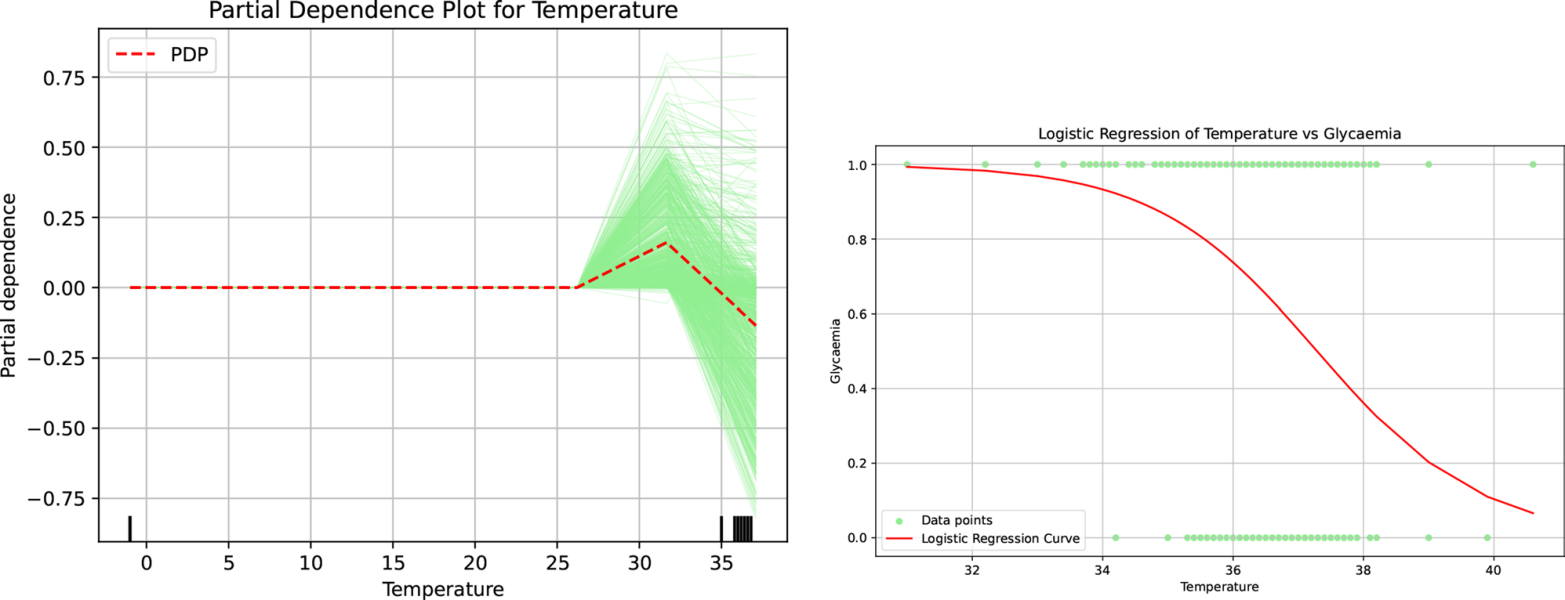
Partial dependence and logistic regression analyses highlighting the role of body temperature in patients’ hypo- or hyperglycemia

Further substantiation of this trend is found in Fig 11. According to the results displayed in the Partial Dependence Plots, logistic regression analysis indicates that patients with a fever exceeding 36$^{\circ}$C face an increased likelihood of being classified as hyperglycemic. This compelling evidence highlights the intertwined nature of these two health indicators and their implications for patient assessment.

According to Fig 9, in patients younger than 87 years old, the third layer of the decision-making model focuses on the chronic renal layer. For this specific demographic, when patients present with a fever alongside chronic renal failure with a serum creatinine level exceeding 1.5 mg/dL, there is a significantly heightened probability of their categorization into the hyperglycemia group. This classification not only reflects a higher level of confidence but also results in a lower Gini index value, indicating more accurate and reliable predictive outcomes.

**Fig. 12:**
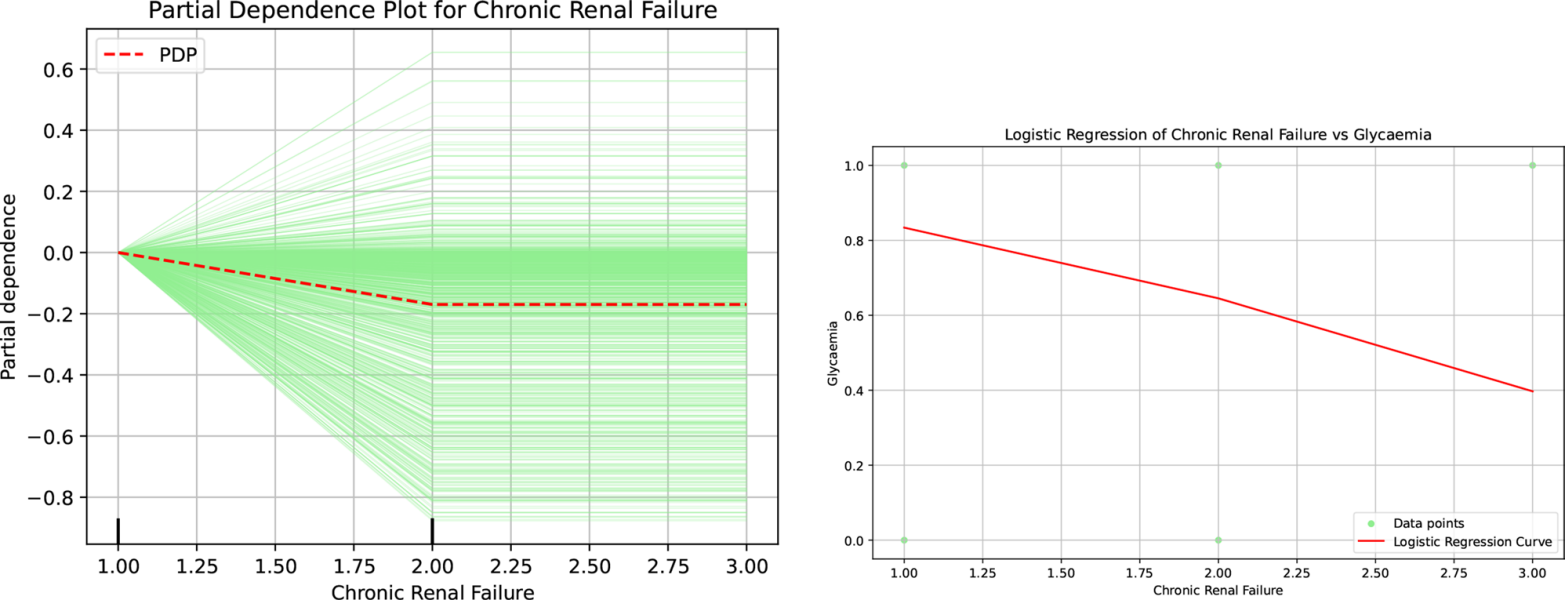
Partial dependence and logistic regression analyses highlighting the role of chronic renal failure in patients’ hypo- or hyperglycemia

By examining the results presented in Fig 12, we can derive important insights related to the evaluation of chronic renal failure. The Partial Dependence Plots specifically illustrates that, as the degree of chronic renal failure escalates—reflected through the increasing values on the horizontal axis—the likelihood of developing hyperglycemia correspondingly increases. This relationship underscores a critical aspect of the disease dynamics and suggests a potential risk factor for patients with varying stages of renal impairment. Moreover, these findings are consistent with the patterns observed in the logistic regression plot, which further corroborates the significant correlation between chronic renal failure and elevated hyperglycemia levels. This analysis emphasizes the importance of monitoring blood glucose levels in patients with chronic renal conditions.

The data illustrated in Fig ?? reveal that platelet levels are an important predictor as its value is assessed across five distinct decision points within the decision tree model. While each decision point utilizes a unique threshold, a comprehensive analysis of these points suggests a clear trend: an increase in platelet levels is associated with an elevated probability of classifying patients as experiencing hypoglycemia. This finding emphasizes the importance of monitoring platelet counts, as they may play a pivotal role in identifying patients at risk for this condition.

**Fig. 13:**
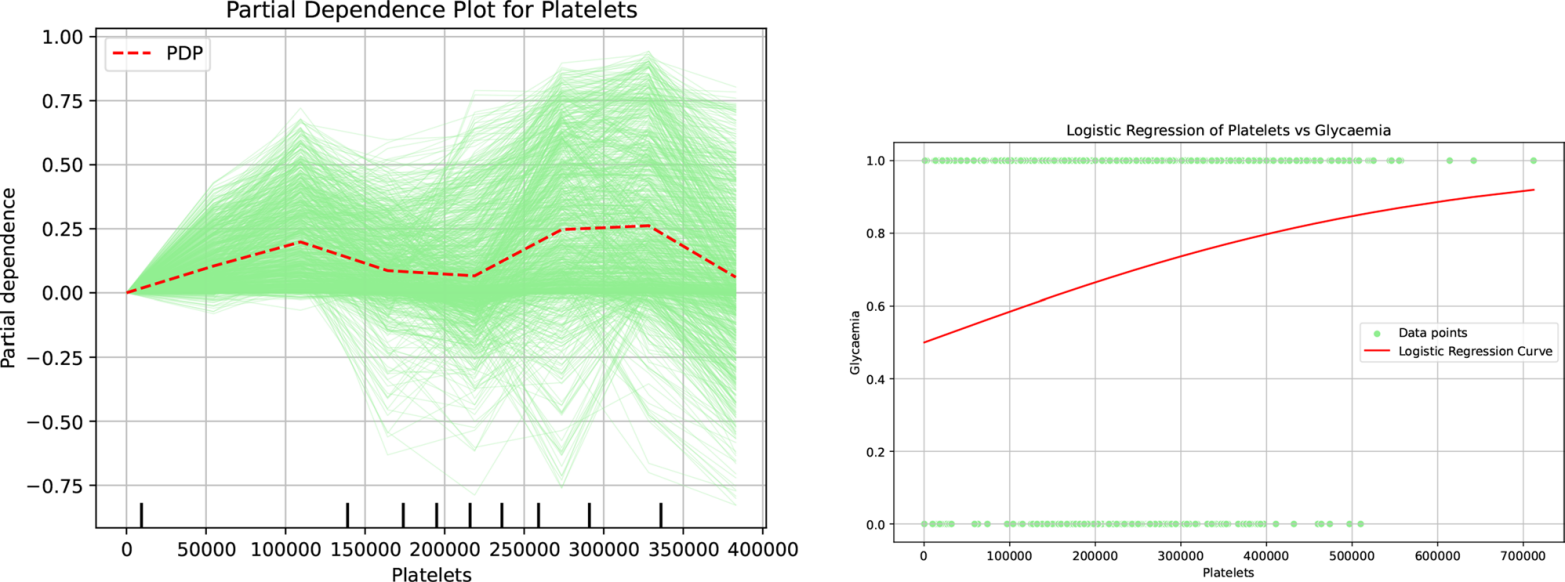
Partial dependence and logistic regression analyses highlighting the role of platelets in patients’ hypo- or hyperglycemia

Figure 13, which illustrates the Partial Dependence Plots of platelets, reveals that while there are some fluctuations in the trends of algorithmic decisions, a consistent pattern emerges: as platelet levels increase, the likelihood of experiencing hypoglycemia rises significantly. This finding is further substantiated by the logistic regression analysis, which indicates that elevated platelet counts correlate with a greater probability of being diagnosed with hypoglycemia. Overall, these results highlight a concerning relationship between platelet levels and hypoglycemic events, suggesting that monitoring platelet counts could be crucial in predicting hypoglycemic episodes.

The final key predictor that warrants further clarification through the application of explainable artificial intelligence (XAI) methods is leukocytes. This predictor is prominently featured at two distinct decision points illustrated in Fig. According to the insights derived from the decision tree, only two cases fall into the hyperglycemia group with a high degree of certainty. In contrast, the remaining cases are classified within the hypoglycemia group, albeit with varying levels of uncertainty. This nuanced distinction highlights the need for a deeper exploration of the role leukocytes play in these classifications.

**Fig. 14:**
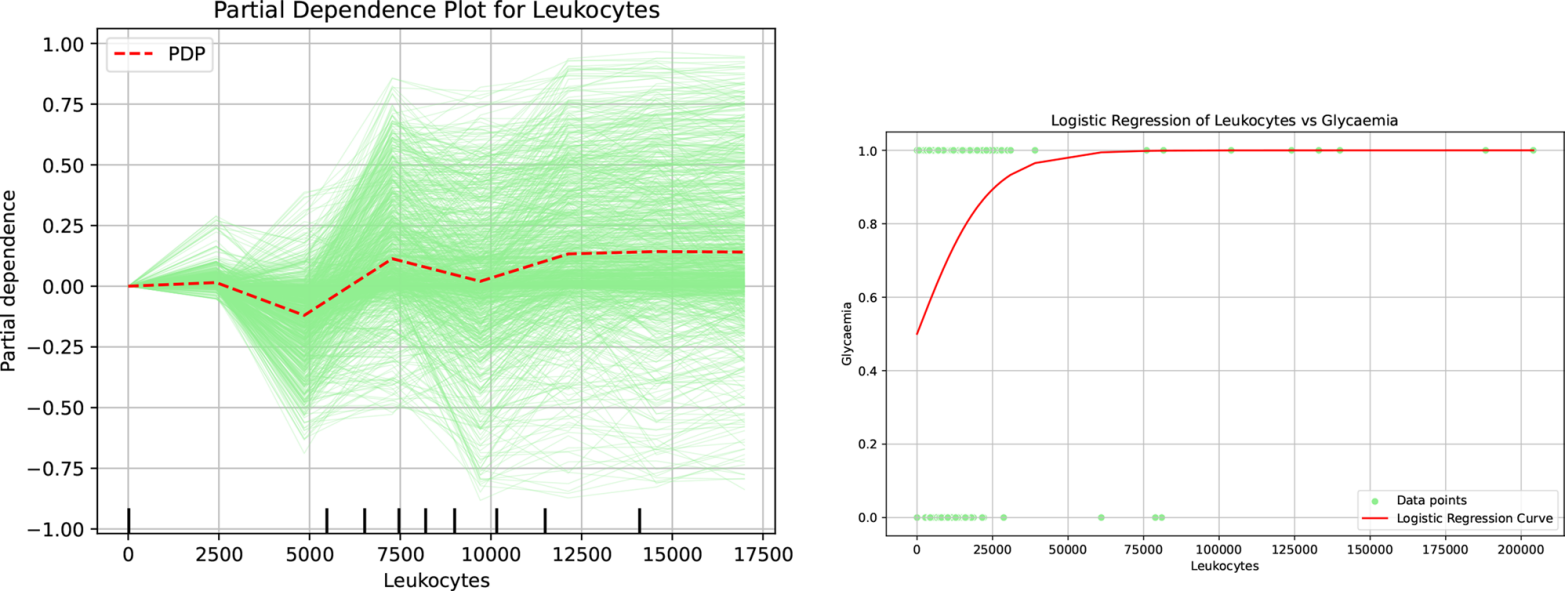
Partial dependence and logistic regression analyses highlighting the role of leukocytes in patients’ hypo- or hyperglycemia

The information derived from Fig 14 demonstrates that an increase in leukocyte levels correlates with a heightened probability of categorizing patients as experiencing hypoglycemia. This finding can be substantiated through an examination of the logistic regression analysis pertaining to the influence of leukocytes on glycemic events in patients. Nonetheless, it is essential to acknowledge a significant valley observed around the leukocyte count of 5000 in the Partial Dependence Plots, indicating the necessity for further investigation to elucidate the underlying factors contributing to this anomaly.

### Interpreting results via domain knowledge

#### Role of age on the patients hypo- or hyperglycemia

The research indicates that advanced age, particularly when accompanied by comorbidities and diminished counter-regulatory mechanisms, such as reduced glucagon or epinephrine secretion, significantly increases the risk of hypoglycemia among elderly patients in a hospital setting Munshi et al. [34]. Among hospitalized patients aged 70 years and older, the incidence of hypoglycemia was observed to be 5.2%. In comparison to those without hypoglycemia, affected patients exhibited a higher prevalence of female gender Kagansky et al. [35]. A study found that frail elderly people aged 65 and older who used insulin or sulfonylureas faced serious low blood sugar events. These events are defined as needing hospitalization, visiting an emergency department, or dying due to blood sugar levels falling below 50 mg/dL. The study included 586 individuals over 33,048 person-years of observation. The rate of these events was higher for insulin users, at 2.76 events per 100 person-years, compared to 1.23 events for sulfonylurea users. The biggest risk factor for these low blood sugar events was being discharged from the hospital within the previous 30 days, which increased the risk by 4.5 times Shorr et al. [36].

#### Role of body temperature on the patients hypo- or hyperglycemia

Both Type 1 and Type 2 diabetes mellitus adversely affect the body’s capacity to regulate core temperature during exposure to extreme heat or cold. In individuals diagnosed with diabetes, the normal physiological responses for heat dissipation—such as increased blood flow to the skin and sweating—are diminished, thereby complicating the body’s ability to tolerate heat stress effectively Kenny et al. [37]. Individuals diagnosed with types 1 and 2 diabetes are reported to be particularly susceptible during periods of extreme heat, as they constitute a disproportionate share of hospitalizations and mortality Schwartz [38]. More recently, diabetes has been linked to impairments in temperature regulation during exposure to thermal stress. In summary, recent studies have established a connection between diabetes and deficits in temperature regulation in response to thermal stress Kenny et al. [37].

#### Role of chronic renal failure on the patients hypo- or hyperglycemia

In patients diagnosed with diabetes who also present advanced CKD, it is essential to customize insulin regimens based on a thorough understanding of individual daily glucose patterns. The implementation of continuous glucose monitoring (CGM) demonstrates considerable potential for enhancing glycemic control in this patient population. This approach may effectively mitigate the risks associated with both hypoglycemia and hyperglycemia Rahhal et al. [39]. Proper glycemic monitoring and individualized control are imperative to prevent hypoglycemia and other glycemic imbalances in patients with type 2 diabetes and concurrent kidney disease. A comprehensive understanding of renal physiology and the pathophysiology of diabetic kidney disease (DKD) is increasingly vital for all medical specialties involved in the management of diabetic patients. Disseminating this knowledge is essential for advancing research initiatives and enhancing the quality of patient care Pecoits-Filho et al. [40]. A reliable assessment of glycemia is essential for effective diabetes management. The kidneys play a crucial role in maintaining glucose homeostasis through processes such as filtration, reabsorption, utilization, and gluconeogenesis. This review underscores the significance of the kidneys in glucose metabolism while examining the advantages, limitations, and existing evidence regarding glycemic markers in individuals with CKD. Particular emphasis is placed on continuous glucose monitoring as an innovative and minimally invasive technique for the assessment of glycemia Hassanein and Shafi [41].

#### Role of platelets on the patients hypo- or hyperglycemia

Platelet activation occurs even in the early stages of impaired glucose metabolism or pre-diabetes, not just in individuals with overt diabetes Santilli et al. [42]. In healthy individuals, hyperglycemia, combined with hyperinsulinemia, can trigger platelet activation and increase tissue factor expression in monocytes. This process promotes a procoagulant and proinflammatory state, which may contribute to acute vascular events and the development of atherosclerosis. Importantly, the responsiveness of platelets to activation by ADP or SFLLRN does not seem to change under conditions of hyperglycemia and hyperinsulinemia Vaidyula et al. [43]. Furthermore, platelet function and thrombogenic potential may actually increase during episodes of hypoglycemia, indicating that both elevated and lowered glucose levels can impact platelet activity Yamamoto et al. [44].

#### Role of leukocytes on the patients hypo- or hyperglycemia

Leukocytosis has demonstrated a strong correlation with the adrenaline response to hypoglycemia Ratter et al. [45]. Resting blood granulocytes and monocytes possess high-affinity facilitative glucose transporters, which likely function as fuel scavengers to support a range of leukocyte activities, encompassing both innate and adaptive immunity, wound healing, tumor surveillance, and tissue remodeling. Modifications in glucose transporter expression under hypoglycemic conditions may signify an autoregulatory mechanism aimed at ensuring an adequate supply of cellular glucose, thereby safeguarding leukocytes from the adverse effects associated with low glucose levels Korgun et al. [46]. Clinical studies indicate that elevated total leukocyte and neutrophil counts, accompanied by diminished eosinophil counts, are significantly associated with diabetic ketoacidosis (DKA) and diabetic ketosis (DK). These findings suggest that leukocyte profiles may provide critical insights regarding hyperglycemic crises and acute infections Xu et al. [47]. In a comprehensive study involving 9697 Chinese adults, a positive correlation was observed between elevated white blood cell (WBC) counts and deteriorating glucose metabolism, including increased body mass index (BMI), larger waist circumference, smoking habits, elevated triglycerides, higher HbA1c levels, and increased postprandial glucose. Conversely, being female and possessing higher levels of high-density lipoprotein (HDL) were related to lower WBC counts. In individuals diagnosed with type 2 diabetes, the duration of the disease did not significantly affect WBC levels. Collectively, an elevated WBC count is independently associated with poorer glucose regulation Jiang et al. [48]

### XAI evaluation framework and empirical analysis

#### Purpose of XAI evaluation

This evaluation affirms that our machine learning models adhere to the standards of clinical validity, transparency, and reproducibility as outlined by CLIX-M and TRIPOD-AI. We are dedicated to not only assessing accuracy but also ensuring that the rationale behind each prediction is communicated in a clear and comprehensible manner for clinicians.

- **Clinical Validation:** Our approach guarantees that model predictions are consistent with established glycemic drivers, such as age, temperature, and renal function, thereby confirming their clinical significance and reproducibility in line with CLIX-M and TRIPOD-AI standards.
- **Trust and Interpretability:** By employing Explainable eXplainable Artificial Intelligence tools, including SHAP and surrogate trees, we validate that the logic of our models aligns with clinical intuition, thus progressing beyond conventional “black box” metrics.
- **Evidence-Based Logic:** We present rigorous evidence demonstrating that our decision-making processes are grounded in established clinical relevance rather than coincidental correlations.

Our ultimate objective is to transcend traditional “black box” metrics and deliver transparent, actionable insights that healthcare providers can confidently rely upon in practical applications.

#### Decision attributes

In this study, we refrained from imposing a priori assumptions on decision attributes. Instead, we employed a data-driven discovery process, allowing the most influential variables to emerge organically from the model’s decision-making logic. By utilizing both global and local eXplainable Artificial Intelligence techniques across the dataset, we initially identified which features consistently influenced predictions before subjecting them to clinical validation.

Through this method, age, body temperature, chronic renal failure, platelet count, and leukocyte count were identified as the most significant attributes in multiple eXplainable Artificial Intelligence analyses. These attributes were then examined in the context of established medical knowledge. Specifically, age is recognized as a factor that influences metabolic regulation and susceptibility to hypoglycemia. Body temperature can indicate acute inflammatory or infectious conditions that affect glucose metabolism. Chronic renal failure impacts insulin clearance and glucose homeostasis. Furthermore, platelet and leukocyte counts act as indicators of physiological stress and inflammation, which have been associated with glycemic instability in hospitalized patients.

By identifying these attributes through model output rather than manual selection, we ensure that the evaluation authentically reflects the Artificial Intelligence’s internal reasoning. This strategy aligns with the CLIX-M principle of transparency, allowing for the verification that the patterns discovered by the model are not only statistically significant but also clinically relevant and actionable for practitioners.

#### Quantitative evaluation

A quantitative evaluation was conducted to assess the consistency and significance of the decision attributes identified through eXplainable Artificial Intelligence across the dataset. Global feature importance was determined utilizing SHAP values aggregated over the entire dataset, serving as a measure of their overall contribution to model predictions. The resulting rankings were subsequently compared to the set of decision attributes identified during the eXplainable Artificial Intelligence-driven discovery process.

As illustrated in the SHAP summary plot (Figure 8) , the variables of age, body temperature, chronic renal failure, platelet count, and leukocyte count consistently emerged as the most significant predictors. Notably, the alignment between these influential features and the decision nodes of the surrogate tree (Figure 9), providing the empirical evidence required for the CLIX-M principle of simulatability by demonstrating that the “black-box” model’s logic is replicable through transparent methods.

Furthermore, the correlation matrix (Figure 6) substantiates the independence and significance of these decision attributes, ensuring the results are not artifacts of multicollinearity. To satisfy the CLIX-M requirement for reproducibility and stability, feature importance rankings were cross-validated across multiple XAI methodologies, including SHAP, global importance rankings (Figure 7), and Partial Dependence Plots (Figure 9, 10, 11, 12 and 13). The observed trends—such as the specific temperature thresholds at 36$^{\circ}$C and 37$^{\circ}$C in Figure 11 and the glycemic risk escalations in Figure 12 remained consistent regardless of data splits, indicating a stable and robust model behavior. This comprehensive mapping of visual evidence to quantitative metrics transitions the study from speculation to a scientifically sound analysis that adheres to the reporting standards.

#### Qualitative evaluation

A qualitative evaluation was conducted to assess the clinical validity of the explanations generated by the XAI methods. This evaluation involved a structured review by practicing clinicians who evaluated the model’s decision paths against standard clinical protocols. Furthermore, a targeted literature review was used to ground the model’s discovered thresholds, such as the 36$^{\circ}$C temperature inflection point in established physiological evidence. These evaluations confirmed that the identified patterns reflect mechanistic clinical relationships rather than coincidental correlations, satisfying the CLIX-M requirement for clinical plausibility.

#### Mapping to CLIX-M

The evaluation of eXplainable Artificial Intelligence conducted in this study was meticulously designed to align with the fundamental principles of the CLIX-M framework, which include transparency, clinical relevance, and reproducibility. Transparency is addressed through the implementation of post-hoc explanation methods that offer both global and local insights into the model’s decision-making process. Clinical relevance is reinforced by identifying decision attributes that align with established medical knowledge and are routinely utilized in clinical practice.

Reproducibility is ensured by consistently applying the same eXplainable Artificial Intelligence methods and evaluation protocols across cross-validation folds, as well as systematically reporting feature importance rankings and representative explanations. To move beyond descriptive adherence, we provide a traceability matrix in Table 2 (Supplementary Materials) that maps each CLIX-M principle directly to the empirical data presented in Figure 8 through 14. Collectively, these components illustrate that the proposed approach adheres to the best practices recommended for explainable Artificial Intelligence in clinical decision support.

#### Limitations

It is important to recognize several limitations associated with the evaluation of eXplainable Artificial Intelligence. The assessment was conducted retrospectively using a single dataset, which may limit the generalizability of the findings to other clinical contexts or populations. Although the provided explanations exhibit clinical plausibility, it is essential to pursue prospective validation that involves clinician engagement to thoroughly evaluate the real-world impact and utility of the proposed methodology.

## Discussion

To the best of our knowledge, this study is the first to focus on predicting glycemic events specifically for patients presenting in the emergency department. Furthermore, it utilizes Explainable eXplainable Artificial Intelligence techniques to demystify the complexities of Artificial Intelligence models and provide valuable insights into the various predictors that contribute to glycemic events in these patients. This approach not only enhances our understanding of the contributing factors but also aids in improving patient outcomes during critical episodes.

In pursuit of the stated objective, we conducted two comprehensive series of analyses aimed at deepening our understanding of the myriad factors that influence glycemic events in patients. This thorough exploration not only examines the complexities of these factors but also evaluates the potential of various Machine Learning methodologies for predicting such events. Our aim is to enable a more informed and nuanced approach to patient care and intervention.

In our analysis, although a direct comparison with elementary classifiers was not undertaken, we observed that our model utilized several significant clinical features, including age, renal function, body temperature, and platelet count. These parameters are consistent with established patterns of glycemic risk. This observation reinforces the model’s reliability and indicates that its predictions are based on meaningful correlations among multiple factors, rather than merely simplistic rules. In future research, we may conduct direct comparisons to more accurately evaluate the added value of the model.

In the initial phase of analysis, referred to as the Wide Feature Set (*I*), we demonstrated that machine learning techniques are capable of accurately classifying patients at risk of hypo- or hyperglycemia, achieving an impressive accuracy rate of up to 74%. This analysis underscored the effectiveness of the Multi-Layer Perceptron and Decision Tree algorithms, which emerged as the top performers. Notably, while these methods excelled, several other approaches also delivered accuracy levels that were remarkably close to 74%. It is important to note that this phase of analysis utilized only 20 predictors, which exhibited a minimal correlation with the target variable, the glycemic event. In the subsequent phase of our analysis, we focused on elucidating the contributions of various predictors by employing a range of eXplainable Artificial Intelligence methods. These included a correlation heatmap matrix, permutation importance, the SHapley Additive exPlanations value method, and surrogate decision trees. The comprehensive assessment of results from these methodologies illuminated several key predictors: age, temperature, platelets, leukocytes, and chronic renal failure emerged as the most significant factors. Moreover, our findings underscored an intriguing aspect of Machine Learning; to optimize accuracy, the models attempted to construct distinct profiles for smaller clusters of participants. While this strategy enhances predictive power, it simultaneously poses a challenge for generalizability across different datasets. It is also important to note that the profiles generated by the machine learning approaches in this study were not easily interpretable by human analysts, adding a layer of complexity to the understanding of the model’s decision-making processes.

In the second phase of our analysis, designated as the Core Feature Set (*II*), we concentrated our efforts on a carefully curated selection of five essential predictors identified during our preliminary exploration. This strategic choice was designed to enhance the generalizability of our findings while simultaneously ensuring that the results remain accessible and easily interpretable for human understanding. During this phase, we employed the same Machine Learning methods as in our initial investigation. Notably, among these methods, Multi-Layer Perceptron and Decision Tree exhibited superior performance compared to the others, with only slight variations in accuracy, ultimately reaching a commendable 70%. This level of performance underscored the efficacy of the Machine Learning techniques utilized in our study.

It should be note that we did not check how well the predicted probabilities match the actual outcomes. Instead, we looked at the overall accuracy of our classifications. We haven’t analyzed how well the predicted risks correspond to the actual occurrence of events. Accurate probabilities are crucial for making informed clinical decisions because they help communicate risks and prioritize treatments. Future research should focus on assessing methods to improve these probabilities, such as using Brier scores and reliability plots. We should also consider recalibration to make our findings more useful in clinical settings.

For the eXplainable Artificial Intelligence component of our analysis, we incorporated the same methodologies as in the first phase, supplemented by Partial Dependence Plots and logistic regression. These additions provided deeper insights into the relationships between our predictors and the outcomes, further enriching our analysis. The comprehensive results derived from our extensive analyses unveiled a limited yet intriguing array of profiles for patients experiencing hypoglycemia and hyperglycemia. Notably, individuals over the age of 87 predominantly fall into the hypoglycemia category, highlighting a crucial age-related trend. In contrast, among the younger population, we identified two distinct profiles that culminate in either hypo- or hyperglycemia. Patients younger than 87 years old, those exhibiting a body temperature exceeding 36 degrees Celsius, those experiencing chronic renal failure with levels greater than 1.5, and those possessing a platelet count below 200,000 are primarily classified as hyperglycemic. Meanwhile, the remaining younger patients who do not meet these criteria are largely categorized within the hypoglycemia group. This nuanced understanding of the different profiles underscores the complex interplay of age, temperature, renal function, and platelet levels in determining glycemic status.

A comprehensive examination of the profiles of patients possesses the remarkable potential to provide invaluable insights for clinicians, enabling them to customize treatments that align closely with each patient’s anticipated glycemic profile. This personalized approach not only enhances individual care but also carries significant implications for public health policymakers. By adopting tailored treatment strategies that are informed by a higher likelihood of success from the outset, we could substantially elevate the effectiveness of therapeutic interventions and optimize resource allocation within the public healthcare system.

It is important to note that the dataset is imbalanced between the hypoglycemia and hyperglycemia classes, with hyperglycemia comprising approximately 30% of the samples. This imbalance can bias predictive models toward the majority class, increasing overall accuracy while compromising sensitivity for hyperglycemia events. To ensure that the model accurately reflects real-world prevalence and to prevent overfitting associated with synthetic oversampling, no class-balancing techniques—such as SMOTE, class weights, or stratified sampling—were employed. Instead, model performance was evaluated using metrics that are particularly sensitive to minority-class predictions, including recall, precision, and F1-score for the hyperglycemia category.

Examining how various factors interact, such as the differential impact of temperature across age groups, is crucial for enhancing the comprehensibility of our models. This study primarily employed decision trees; however, we did not investigate alternative methodologies that could elucidate these complex interactions. We intend to explore these interactions in future research, as this may yield deeper insights into the combined effects of various factors on outcomes and contribute to the overall clarity of our models.

Nonetheless, the findings presented here should be approached with discernment, as the relatively modest sample size may have significantly compromised their generalizability. It is important to acknowledge that numerous other factors, not included in our database, could have influenced the results obtained, such as participants’ diet, ethnicity, and lifestyle. This limitation suggests a diminished predictive power of the algorithms employed. Despite this constraint, our study lays an essential groundwork for future research endeavors, advocating the pragmatic application of similar models. Such a system could empower healthcare professionals and service providers by allowing them to input relevant metrics, thus paving the way for timely and tailored therapeutic interventions that cater to individual patient needs. Our study used data from 2018 to 2019. We recognize that diabetes management has changed since then. New medications like GLP-1 receptor agonists and SGLT2 inhibitors are now more common. Updated guidelines for blood sugar goals exist, and the COVID-19 pandemic has also influenced how care is provided. These changes may affect the frequency and characteristics of glycemic events, so the performance of our model may differ for more recent patient cohorts. We suggest retraining or updating the model with new data before using it and regularly recalibrating it as practices change. However, the identified risk factors—age, kidney function, body temperature, and blood test results—are still important and likely continue to help in assessing patient risk.

The models we developed achieved accuracies between 70 and 74%. However, accuracy alone does not fully reflect how useful these models are in a clinical setting. An error rate of 26%-30% needs to be considered alongside how often the events occur and the possible clinical effects of different types of errors. The importance of these errors depends on the number of false negatives (missed hyperglycemia) compared to false positives (incorrectly identified hyperglycemia). We suggest using the model as a helpful tool, not as a replacement for a clinician’s judgment. In practice, the model can help identify patients needing closer monitoring or prompt testing, like checking blood sugar levels, rather than starting treatment without a clinician’s review. If patient safety is a priority, you may want to choose thresholds that focus on catching more cases, even if that results in more false alarms. We also included analyses to explain how the model makes decisions to ensure it aligns with established clinical practices. The most influential features in the model closely match the factors that clinicians find important for assessing glycaemic risk, and experts have reviewed these to confirm their impact. This builds confidence in the model’s ability to work in a way that makes sense physiologically and is trustworthy in a clinical setting, highlighting its role as a support for clinical judgment rather than a replacement.

In this study, we employed various methods, including Pearson correlation heatmaps, partial dependence plots (PDPs), logistic regression, SHAP, surrogate decision trees, and decision-tree feature-importance measures, to enhance the interpretability of our predictive model. Each of these approaches provides associational insights, highlighting features that are correlated with the model’s predictions; however, it is important to note that they do not establish causal relationships. For example, the feature importance derived from decision trees illustrates which variables significantly contribute to the model’s predictive accuracy, but it does not imply that altering these features would necessarily affect the outcomes. Additionally, we acknowledge certain limitations, such as potential biases inherent in the dataset, the linearity assumptions associated with logistic regression, the potential reduction in fidelity from surrogate tree approximations, and the assumptions of feature independence in PDP analysis. It is crucial for clinicians to interpret the findings derived from these explainable artificial intelligence (XAI) methods with care, as they reflect patterns generated by the model rather than definitive cause-and-effect relationships.

Numerous diabetes risk scores, including those that predict diabetic ketoacidosis (DKA) and episodes of hypoglycemia, typically emphasize the management of patients in hospital or clinical settings. These existing scores often rely on data that is not routinely gathered in the emergency department (ED). Consequently, our model was not directly compared to these established scores. Instead, our approach uses readily available data to predict both low- and high-blood-sugar events, offering a novel and adaptable methodology for early risk identification in emergency care. Future research could evaluate our model’s performance against established scores in similar patient populations.

Our primary objective was to demonstrate the application of a specific methodology to real-world data. Leveraging a larger sample size may not only yield more definitive conclusions but also facilitate the identification of instances where the algorithm excels in providing accurate predictions, as well as those where it may fall short. Additionally, this methodological approach holds promise for future investigations, opening the door to the exploration of a wider array of variables for their predictive significance, with the ultimate aspiration of refining the accuracy of our predictions.

## Conclusion

The results of the study reveal that supervised Machine Learning can yield significant insights into patients’ glycemic events, taking into account various individual characteristics. Furthermore, this research contributes essential understanding of the elements that enhance the accurate identification and interpretation of these events. By employing a rigorous methodology, the study illuminates the complex interplay between patients’ glycemic events and a range of other influencing factors, offering a comprehensive perspective on this critical aspect of patient health management.

In the context of our analysis involving the eXplainable Artificial Intelligence component, we implemented a range of techniques to enhance our understanding of the relationships between predictors and outcomes. The findings revealed distinct profiles for patients experiencing hypoglycemia and hyperglycemia. Notably, individuals aged 87 years and older predominantly fell into the hypoglycemia category. Conversely, younger patients, specifically those under 87 years of age, exhibited two profiles associated with hypo- or hyperglycemia. Those under 87 with a body temperature exceeding 36 degrees Celsius(upon arrival to the emergency section), chronic renal failure (defined by creatinine levels above 1.5), and a platelet count below 200,000 were classified as hyperglycemic, while the remaining younger patients were primarily categorized as hypoglycemic. This highlights the complex interactions among age, body temperature, renal function, and platelet levels in influencing glycemic status.

To further substantiate our findings, it is essential to conduct additional research with a broader and more diverse sample. This model holds the potential to strategically guide healthcare professionals in formulating the most effective treatment approaches for individuals living with diabetes. The outcomes of this research could also have significant implications for public healthcare programs and policies, enabling a more targeted and efficient allocation of resources to improve patient outcomes and overall community health Rotbei.

## Electronic supplementary material

Below is the link to the electronic supplementary material.

Supplementary Material 1

## Data Availability

The datasets used and analyzed during the current study are available from the corresponding author on reasonable request.

## Abbreviations

T1DM: Type 1 Diabetes Mellitus
T2DM: Type 2 Diabetes Mellitus
DM: Diabetes Mellitus
ML: Machine Learning
AdaBoost: Adaptive Boosting
MLP: Multi-Layer Perceptron
LR: Logistic Regression
PDP: Partial Dependence Plots
AI: Artificial Intelligence
XAI: eXplainable Artificial Intelligence
SHAP: SHapley Additive exPlanations
GDP: Gross Domestic Product
ED: Emergency Departments
CKD: Chronic Kidney Disease

## References

[1] CDC. Centers for disease control and prevention. 2024-2025. https://data.cdc.gov/.

[2] OECD. Organisation for economic co-operation and development. 2024-2025. https://www.oecd.org/spain/.

[3] Commission E, Eurostat. HEDIC – health expenditures by diseases and conditions – 2016. Publications Office; 2016.

[4] Annuzzi G, Apicella A, Arpaia P, Bozzetto L, Criscuolo S, De Benedetto E, et al. Exploring nutritional influence on blood glucose forecasting for type 1 diabetes using explainable ai. IEEE J Biomed Health Inf. 2024;28(5):3123–33. 10.1109/JBHI.2023.3348334 38157465

[5] Sapra A, Bhandari P. Diabetes mellitus. 2021 sep 18. StatPearls [Internet]. Treasure Island (FL): StatPearls Publishing; 2022.

[6] Hoyos J, Villa-Tamayo MF, Builes-Montaño CE, Ramirez-Rincón A, Godoy JL, Garcia-Tirado J, et al. Identifiability of control-oriented glucose-insulin linear models: review and analysis. IEEE Access. 2021;9:69173–88.

[7] Almazán MS, Carretero TM, Ramón SS, Bravo MTJ, Soto CC. Estudio descriptivo de las complicaciones agudas diabéticas atendidas en un servicio de urgencias hospitalario. Emerg Rev Soc Esp Med Urgenc Emerg. 2017;29(4):245–48. 28825279

[8] Khodaei MJ, Candelino N, Mehrvarz A, Jalili N. Physiological closed-loop control (pclc) systems: review of a modern frontier in automation. IEEE Access. 2020;8:23965–4005.

[9] Annuzzi G, Arpaia P, Bozzetto L, Criscuolo S, Giugliano S, Pesola M. Assessing the features on blood glucose level prediction in type 1 diabetes patients through explainable artificial intelligence. 2023 IEEE Int Conf Metrol Ext Reality, Artif Intel Neural Eng (MetroXRAINE). 2023;278–83. 10.1109/MetroXRAINE58569.2023.10405831.

[10] Annuzzi G, Arpaia P, Bozzetto L, Criscuolo S, De Benedetto E, Pesola M. Explainable ai assessment of meal-related features impact in predicting basal insulin for type i diabetes. 2024 IEEE 8th Forum Res Technol Soc Ind Innov (RTSI). 2024;396–401. 10.1109/RTSI61910.2024.10761239.

[11] Botta A, Rotbei S, Zinno S, Ventre G. Cyber security of robots: a comprehensive survey. Intell Syst Appl. 2023;18:200237.

[12] Peral J, Gil D, Rotbei S, Amador S, Guerrero M, Moradi H. A machine learning and integration based architecture for cognitive disorder detection used for early autism screening. Electronics. 2020;9(3):516.

[13] Rotbei S, Mocerino GE, Haleem MS, Pecchia L, Botta A. Frequency and uncertainty driven deep learning approach to segment electrocardiogram signals for effective heart parameters estimation. 2024 IEEE EMBS Int Conf Biomed Health Inf (BHI). 2024;1–8. 10.1109/BHI62660.2024.10913758.

[14] Rotbei S, Napolitano L, Zinno S, Verze P, Botta A. Predicting patient sexual function after prostate surgery using machine learning. 2023 IEEE Symp Comput Commun (ISCC). 2023;1–6. IEEE.

[15] Rotbei S, Tseng WH, Merino-Barbancho B, Haleem MS, Montesinos L, Pecchia L, et al. Evaluating impact of movement on diabetes via artificial intelligence and smart devices systematic literature review. Expert Syst Appl. 2024;125058.

[16] Rotbei S, Soler PM, Merino-Barbancho B, Tourab H, Corbatón Anchuelo A, García LP, et al. Prediction of glycemic event in emergency section patients using machine learning. 2024 IEEE Int Conf E-Health Netw, Application Serv (HealthCom). 2024;1–4. 10.1109/HealthCom60970.2024.10880819.

[17] Zhu T, Uduku C, Li K, Herrero P, Oliver N, Georgiou P. Enhancing self-management in type 1 diabetes with wearables and deep learning. NPJ Digit Med. 2022;5(1):78. 35760819 10.1038/s41746-022-00626-5 PMC9237131

[18] Jacobs PG, Herrero P, Facchinetti A, Vehi J, Kovatchev B, Breton MD, et al. Artificial intelligence and machine learning for improving glycemic control in diabetes: best practices, pitfalls, and opportunities. IEEE Rev Biomed Eng. 2023;17:19–41. 10.1109/RBME.2023.3331297 37943654

[19] Nguyen M, Jankovic I, Kalesinskas L, Baiocchi M, Chen JH. Machine learning for initial insulin estimation in hospitalized patients. J Am Med Inf Assoc. 2021;28(10):2212–19. 10.1093/jamia/ocab099 PMC8449602 34279615

[20] De Bois M, Yacoubi MAE, Ammi M. Glyfe: review and benchmark of personalized glucose predictive models in type 1 diabetes. Med Biol Eng Comput. 2022;60(1):1–17. 34751904 10.1007/s11517-021-02437-4

[21] Guzman Gómez GE, Agredo LEB, Martínez V, Leiva OFB. Application of artificial intelligence techniques for the estimation of basal insulin in patients with type i diabetes. Int J Endocrinol. 2020;2020(1):7326073. 33204261 10.1155/2020/7326073 PMC7655245

[22] Antoniadi AM, Du Y, Guendouz Y, Wei L, Mazo C, Becker BA, et al. Current challenges and future opportunities for xai in machine learning-based clinical decision support systems: a systematic review. Appl Sci. 2021;11(11):5088.

[23] Markus AF, Kors JA, Rijnbeek PR. The role of explainability in creating trustworthy artificial intelligence for health care: a comprehensive survey of the terminology, design choices, and evaluation strategies. J Retailing Biomed Inf. 2021;113:103655. 10.1016/j.jbi.2020.103655 33309898

[24] Josse J, Chen JM, Prost N, Varoquaux G, Scornet E. On the consistency of supervised learning with missing values. Stat Papers. 2024;65(9):5447–79.

[25] Urtnasan E, Joo EY, Lee KH. Ai-enabled algorithm for automatic classification of sleep disorders based on single-lead electrocardiogram. Diagnostics. 2021;11(11):2054. 10.3390/diagnostics11112054 PMC8620146 34829400

[26] Chen R-C, Dewi C, Huang S-W, Caraka RE. Selecting critical features for data classification based on machine learning methods. J Educ Chang Big Data. 2020;7(1):52.

[27] Altmann A, Toloşi L, Sander O, Lengauer T. Permutation importance: a corrected feature importance measure. Bioinformatics. 2010;26(10):1340–47. 20385727 10.1093/bioinformatics/btq134

[28] Kijsipongse E, U-Ruekolan S, Ngamphiw C, Tongsima S. Efficient large pearson correlation matrix computing using hybrid mpi/cuda. 2011 Eighth Int Joint Conf Comput Sci Softw Eng (JCSSE). 2011;237–41.

[29] Salih AM, Raisi-Estabragh Z, Galazzo IB, Radeva P, Petersen SE, Lekadir K, et al. A perspective on explainable artificial intelligence methods: Shap and lime. Adv Intell Syst. 2025;7(1):2400304.

[30] Li Z. Extracting spatial effects from machine learning model using local interpretation method: an example of shap and xgboost. Comput, Environ Urban Syst. 2022;96:101845.

[31] Di Castro F, Bertini E. Surrogate decision tree visualization. IUI Workshops. 2019.

[32] Pettorruso M, Guidotti R, d’Andrea G, De Risio L, D’Andrea A, Chiappini S, et al. Predicting outcome with intranasal esketamine treatment: a machine-learning, three-month study in treatment-resistant depression (esk-learning). Psychiatry Res. 2023;327:115378. 37574600 10.1016/j.psychres.2023.115378

[33] Franklin J. The elements of statistical learning: data mining, inference and prediction. The Math Intelligencer. 2005;27(2):83–85.

[34] Munshi MN, Segal AR, Suhl E, Staum E, Desrochers L, Sternthal A, et al. Frequent hypoglycemia among elderly patients with poor glycemic control. Archives Intern Med. 2011;171(4):362–64. 10.1001/archinternmed.2010.539 PMC4123960 21357814

[35] Kagansky N, Levy S, Rimon E, Cojocaru L, Fridman A, Ozer Z, et al. Hypoglycemia as a predictor of mortality in hospitalized elderly patients. Archives Intern Med. 2003;163(15):1825–29. 10.1001/archinte.163.15.1825 12912719

[36] Shorr RI, Ray WA, Daugherty JR, Griffin MR. Incidence and risk factors for serious hypoglycemia in older persons using insulin or sulfonylureas. Archives Intern Med. 1997;157(15):1681–86. 9250229

[37] Kenny GP, Sigal RJ, McGinn R. Body temperature regulation in diabetes. Temperature. 2016;3(1):119–45. 10.1080/23328940.2015.1131506 PMC4861190 27227101

[38] Schwartz J. Who is sensitive to extremes of temperature?: A case-only analysis. Epidemiology. 2005;16(1):67–72. 15613947 10.1097/01.ede.0000147114.25957.71

[39] Rahhal M-N, Gharaibeh NE, Rahimi L, Ismail-Beigi F. Disturbances in insulin–glucose metabolism in patients with advanced renal disease with and without diabetes. J Clin Endocr Metab. 2019;104(11):4949–66. 31162534 10.1210/jc.2019-00286

[40] Pecoits-Filho R, Abensur H, Betônico CC, Machado AD, Parente EB, Queiroz M, et al. Interactions between kidney disease and diabetes: dangerous liaisons. Diabetology Metabolic Syndr. 2016;8(1):50. 10.1186/s13098-016-0159-z PMC4964290 27471550

[41] Hassanein M, Shafi T. Assessment of glycemia in chronic kidney disease. BMC Med. 2022;20(1):117. 35414081 10.1186/s12916-022-02316-1 PMC9006428

[42] Santilli F, Simeone P, Liani R, Davì G. Platelets and diabetes mellitus. Prostaglandins Other Lipid Mediators. 2015;120:28–39. 25986598 10.1016/j.prostaglandins.2015.05.002

[43] Vaidyula VR, Boden G, Rao AK. Platelet and monocyte activation by hyperglycemia and hyperinsulinemia in healthy subjects. Platelets. 2006;17(8):577–85. 17127486 10.1080/09537100600760814

[44] Yamamoto K, Ito T, Nagasato T, Shinnakasu A, Kurano M, Arimura A, et al. Effects of glycemic control and hypoglycemia on thrombus formation assessed using automated microchip flow chamber system: an exploratory observational study. Thromb J. 2019;17(1):17. 31496922 10.1186/s12959-019-0206-8 PMC6717975

[45] Ratter JM, Rooijackers HM, Tack CJ, Hijmans AG, Netea MG, De Galan BE, et al. Proinflammatory effects of hypoglycemia in humans with or without diabetes. Diabetes. 2017;66(4):1052–61. 28115398 10.2337/db16-1091

[46] Korgun E, Demir R, Sedlmayr P, Desoye G, Arikan G, Puerstner P, et al. Sustained hypoglycemia affects glucose transporter expression of human blood leukocytes. Blood Cells Mol Dis. 2002;28(2):152–59. 12064911 10.1006/bcmd.2002.0504

[47] Xu W, Wu H-F, Ma S-G, Bai F, Hu W, Jin Y, et al. Correlation between peripheral white blood cell counts and hyperglycemic emergencies. Int J Med Sci. 2013;10(6):758. 23630441 10.7150/ijms.6155 PMC3638300

[48] Jiang H, Yan W-H, Li C-J, Wang A-P, Dou J-T, Mu Y-M. Elevated white blood cell count is associated with higher risk of glucose metabolism disorders in middle-aged and elderly Chinese people. Int J Environ Res Public Health. 2014;11(5):5497–509. 24852600 10.3390/ijerph110505497 PMC4053882

