# Supplementary material for: Exploring the potential of XAI methods in generating clinically meaningful explanations for glycemia prediction in diabetes patients
Source: BMC Med Inform Decis Mak. 2026 Mar 14;26:135. doi: 10.1186/s12911-026-03420-5 (PMC13101347; doi:10.1186/s12911-026-03420-5)
Supplement: Supplementary file 1 — Supplementary Material 1 [file 12911_2026_3420_MOESM1_ESM.pdf]

1 Supplementary Material

1.1 Clinician-Informed XAI Evaluation Checklist with Metrics (CLIX-M)

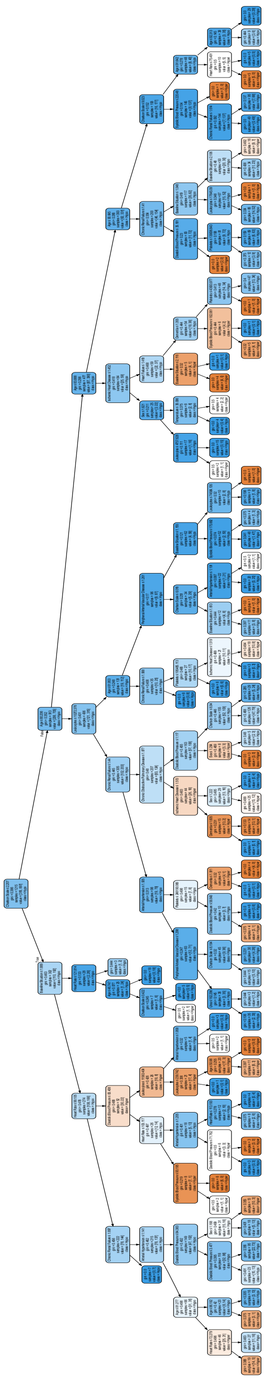

Fig. 1 Surrogate decision tree used to explain black-box model behavior. The downward arrow represents the 'True' branch, while the upward arrow represents the 'False' branch.

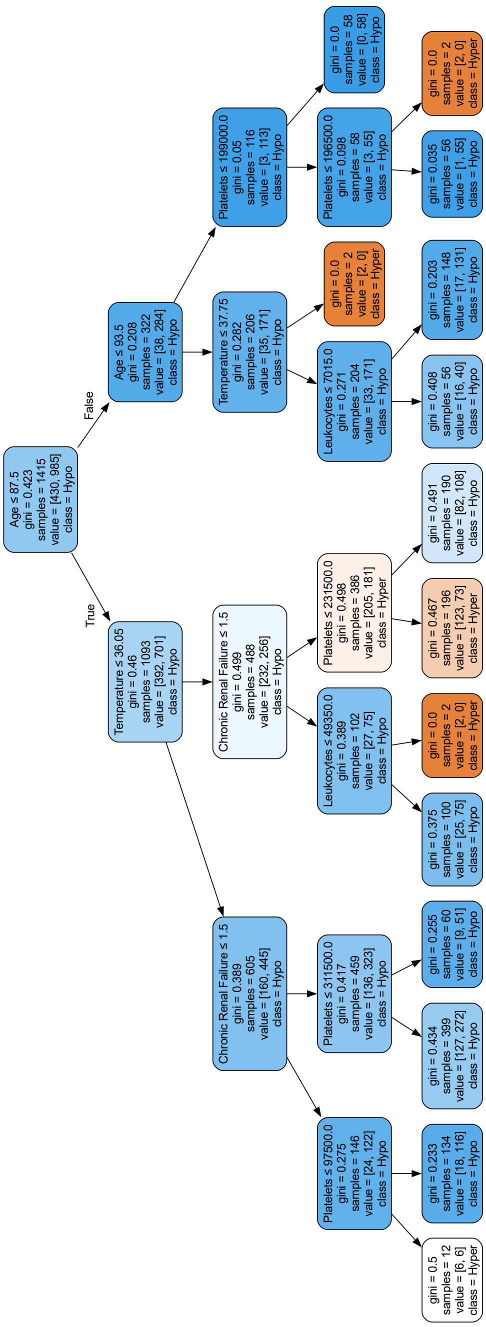

Fig. 2 Surrogate decision tree used to explain black-box model behavior with key predictors

Table 1 TRIPOD Checklist (Transparent Reporting of a Multivariable Prediction Model for Individual Prognosis or Diagnosis) This table summarizes how each TRIPOD item is addressed in the manuscript and supplementary materials.

Section	Item	Reported	Details / Where Addressed
**Title & Abstract**	Title identifies prediction model, outcome, and population	X	Specifies glycemia prediction in diabetes using AI-based models
	Abstract includes objectives, design, setting, participants, predictors, outcome, and key results	X	Briefly summarizes dataset, ML methods, results, and XAI components
**Introduction**	Medical/clinical background and rationale	X	Describes diabetes complications and relevance of glycemic event prediction in ED settings
	Clear study objective	X	Aim is to predict hypoglycemia/hyperglycemia events with interpretable ML
**Methods**	Source of data and data origin	X	Multicenter retrospective dataset from 11 hospitals across Spain
	Key dates (data collection, follow-up)	X	July 1, 2018 to July 1, 2019
	Study setting	X	Emergency departments (EDs) in Spain
	Inclusion criteria	X	Adults (> 18 years), diagnosed with T1DM or T2DM, ED visit with glycemic event
	Exclusion criteria	X	Absence of T1DM/T2DM diagnosis or incomplete ED encounter
	Outcome definition and assessment	X	Binary outcome: hypoglycemia vs. hyperglycemia based on ED-measured glucose levels
	List and definition of predictors	X	55 predictors collected; reduced to 20 after filtering; final 5 according to feature importance
	Sample size explanation	X	1,415 encounters initially; 1,215 included in modelling after quality checks
	Handling of missing data	X	Low missingness; imputed using value −1 to represent “unknown” state
	Prediction model(s) specified	X	Eight ML algorithms trained with hyperparameter tuning (GridSearchCV)
	Model validation	X	Internal validation using stratified 5-fold cross-validation
	Performance measures	X	Accuracy, Precision, Recall, F1-score reported for all models
	Feature selection and dimensionality reduction	X	Correlation filtering, model-based feature importance, and SHAP analysis
	Model interpretability methods	X	SHAP values, Partial Dependence Plots (PDP), surrogate decision trees
**Results**	Participant flow and numbers	X	Flow from 1,415 initial records to 1,215 complete cases used for modelling
	Model performance	X	AdaBoost and MLP achieved highest performance (74% accuracy)
	Predictor importance	X	Interpretable rankings and clinical explanations provided for key predictors
	Visual outputs	X	SHAP summary plots, decision trees, and correlation matrices included
**Discussion**	Interpretation of prediction model performance	X	Strengths, signal patterns, and clinical implications discussed
	Study limitations	X	Notes generalizability limits, retrospective design, and interpretability constraints
**Other Information**	Ethical approval	X	Approved by the Ethics Committee of Hospital Clínico San Carlos (May 6, 2022)
	Funding and conflicts of interest	X	Not explicitly stated in the present excerpt
	Availability of model or code	X	Uses widely available algorithms; implementation described for reproducibility
	External validation	X	No external dataset used; only internal 5-fold cross-validation performed
	Calibration assessment	X	Not conducted

Table 2 CLIX-M Checklist: Evaluation of Explainable AI for Glycemia Prediction in Emergency Department Patients. * **Phase**: D = XAI development; E = Evaluation of XAI in clinical settings.
† **Section**: M = Methods; R = Results; D = Discussion.
‡ TP = True Positive, TN = True Negative, FP = False Positive, FN = False Negative.

No.	Checklist Item	Item Details	Phase*	Section†
**Clinical Attributes**				
1	**Purpose**	The study aims to identify patients at risk of hypoglycemia or hyperglycemia using routinely collected emergency department data from 11 hospitals in Spain.	E	M, R, D
2	**Domain Relevance**	R: Literature review confirms that the findings are clinically relevant. D: Experts in emergency medicine and diabetology rated all considered variables as clinically meaningful.	E	R, D
3	**Coherence**	R: Analyses revealed informative patient profiles for glycemic events. Patients over 87 years old predominantly experienced hypoglycemia, indicating an age-related pattern. Among adults younger than 87, hyperglycemia was associated with body temperature $>$ 36°C, chronic renal failure (creatinine $>$ 1.5 mg/dL), and platelet counts $<$ 200,000/μL. D: These patterns highlight the interplay of age, temperature, renal function, and platelet levels in differentiating glycemic status.	D, E	R, D
4	**Actionability**	R: The top predictive features were judged by clinicians as clinically actionable and aligned with existing literature. D: The study's methodological and dataset limitations warrant cautious interpretation before clinical adoption.	D, E	R, D
**Decision Attributes**				
5	**Correctness**	R: Findings are consistent with domain knowledge and verified by clinical experts. Multiple XAI and ML methods produced similar feature rankings, increasing reliability. D: The dataset is relatively small and not fully representative, limiting generalizability.	D, E	R, D
6	**Confidence**	M: The rationale for all modelling and XAI methods is fully described to justify appropriateness. R: Multiple approaches produced convergent, literature-consistent results, supporting reliability.	D, E	M, R
7	**Consistency**	R: All applied ML and XAI approaches yielded similar conclusions, supporting robustness. D: High agreement across methods reinforces the internal consistency of the findings.	D, E	R, D
8	**XAI Robustness**	R: Agreement across SHAP, PDP, and surrogate-tree explanations, combined with expert validation, supports strong robustness. D: Despite ensemble XAI improving stability, dataset constraints still limit broader generalizability.	D, E	R, D
9	**Causal Validity**	R: Observed causal tendencies were cross-checked with established clinical knowledge, ensuring coherent and biologically plausible interpretations.	D, E	R
**Machine Attributes**				
10	**Narrative Reasoning**	R, D: All findings are explained through detailed, step-by-step clinical and computational reasoning.	D, E	R, D
11	**Bias and Fairness**	R: Evaluation showed no significant evidence of bias by age or sex. Class imbalance reflected real-world prevalence, and the employed ML methods handled imbalance adequately.	D, E	R
12	**Model Troubleshooting**	R: Key predictors were examined in correctly classified samples. Thresholds and data distribution patterns were reviewed to guide future model refinement.	D, E	R
13	**Interpretation**	R, D: Model decisions are supported by clinically meaningful, domain-consistent features with agreement across XAI methods and expert validation. Results align with previous literature and demonstrate transparency and reliability for audit purposes.	D, E	R, D
14	**XAI Limitations**	The limitations of the methodology and the relatively small dataset require cautious interpretation in clinical practice.	D, E	D
